# Labile carbon drives synergistic improvements in *Astragalus membranaceus* yield and quality under chemical fertilizer reduction combined with organic amendments

**DOI:** 10.3389/fpls.2026.1809778

**Published:** 2026-05-01

**Authors:** Zhiwang Rao, Xia He, Xinyue Wang, Min Yan, Zhiping Yang, Qiang Zhang, Junling Guo

**Affiliations:** 1 College of Resource & Environment, Shanxi Agricultural University, Taiyuan, Shanxi, China; 2 Institute of Eco-environment and Industrial Technology, Shanxi Agricultural University, and Soil Health Laboratory in Shanxi Province, Taiyuan, Shanxi, China

**Keywords:** active constituent, chemical fertilizer reduction, Hengshan *Astragalus membranaceus*, medicinal plants, organic amendments, soil labile carbon pool

## Abstract

Over-fertilization and soil organic matter deficiency are common problems in *Astragalus membranaceus (A. membranaceus)* cultivation, severely limiting its yield and quality. This study conducted a field experiment to investigate the effects and underlying mechanisms of chemical fertilizer reduction combined with organic amendments (CFR+OA) on the yield and quality of *A. membranaceus*. The objective was to evaluate the practical feasibility of CFR+OA technology for *A. membranaceus* and to identify the key factors affecting its yield and quality by analyzing basic soil physicochemical properties, labile carbon fractions, and enzyme activities. Five treatments were tested: (i) farmer practice (FP), (ii) chemical fertilizer reduction (CFR), and three CFR+OA combinations—(iii) organic fertilizer (OF), (iv) bio-organic fertilizer (BOF), and (v) vermicompost (Vm). Results showed that CFR+OA treatments enhanced the yield, quality, and profit of *A. membranaceus*, with BOF exhibiting superior performance. Compared with FP, BOF treatment significantly increased root dry weight and active constituent yield (ACY) by 9.86% and 43.31%, respectively, and increased profit by 1,897.23 USD·ha^-^¹. In addition, CFR+OA treatments significantly improved soil properties. Compared with FP, BOF treatment markedly increased soil available nutrients (AP, AK), labile carbon fractions (POC, ROC, DOC, MBC), and enzyme activities (S-UE, S-SC, S-ALP) by more than 15%, with AP, POC, MBC, and S-ALP increasing by over 50%. The Random Forest analysis identified TN, AK, POC, MBC, and S-SC as key common drivers that significantly influence the RDW and ACY of *A. membranaceus* (*P* < 0.05). Among these, POC was the only factor showing a highly significant positive effect on both yield and ACY (*P* < 0.01). This result indicates that the soil labile carbon pool, dominated by POC, plays a critical role in regulating the yield and quality of *A. membranaceus*. In conclusion, this study demonstrates that CFR+OA is validated as a fertilization technology for Hengshan *A. membranaceus* cultivation, achieving the goal of “carbon-fertilizes-soil, carbon-boosts-yield”. CFR+BOF effectively improves soil properties and enhances the yield and quality of *A. membranaceus*, demonstrating great potential for widespread application.

## 1 Introduction

Medicinal plants, as a vital resource connecting traditional medicine with modern healthcare systems, play a crucial role in protecting human health and supporting economic development (Jamshidi-Kia et al., 2018). With the rising global demand for natural medicinal resources, optimizing cultivation and management practices to achieve high quality and high yield has become a key challenge for the industry’s development (Jiang et al., 2022). Huangqi refers to the dried root of *Astragalus membranaceus* var. *mongholicus* (Bunge) P.K. Hsiao or *Astragalus membranaceus* (Fisch.) Bge., both belonging to the Fabaceae family (Bi et al., 2020; Liu et al., 2022). Globally, the plant is widely distributed across China, Russia, Kazakhstan, and Mongolia (Li et al., 2025).

Often regarded as the King of Qi Tonics in Traditional Chinese Medicine, it has been traditionally used to tonify Qi, strengthen the spleen, and benefit the lungs (Zhang et al., 2024c). Its major active constituents include flavonoids, saponins, and polysaccharides, which exhibit diverse pharmacological activities, including immunomodulatory, anti-inflammatory, anti-fatigue, anti-aging, hypolipidemic, and antitumor effects (Dong et al., 2023; Liu et al., 2024b, 2025). In China, it is widely growing across provinces, including Inner Mongolia, Shanxi, Shaanxi, Gansu, Hebei, Ningxia, and Heilongjiang (Li et al., 2024; Wang et al., 2024). Hengshan *A. membranaceus* is a specialty of the Hengshan region in northern Shanxi Province, China. The region’s cool climate and unique sandy soils, developed from granite gneiss, provide optimal growing conditions, contributing to its superior quality (Dong et al., 2024; Hou et al., 2025a). Owing to its superior quality, Hengshan *A. membranaceus* is well known in domestic markets and exported worldwide.

The quality of medicinal plants is determined by genetic background and climatic conditions, but is also significantly modulated by soil quality and nutrient supply (Su et al., 2023). As a pivotal indicator of soil health and fertility, soil organic carbon (SOC) directly influences crop growth and the formation of quality traits through its content and activity (He et al., 2022b). Compared with SOC, soil labile carbon fractions exhibit greater sensitivity to changes in soil physicochemical properties, environmental factors, and fertilization regimes, making them important indicators of soil quality variation. Currently, the main soil labile carbon fractions of interest include particulate organic carbon (POC), readily oxidizable organic carbon (ROC), dissolved organic carbon (DOC), and microbial biomass carbon (MBC) (Dai et al., 2021b; Anandakumar et al., 2022).

Chemical fertilizer reduction combined with organic amendments (CFR+OA) is widely recognized as an important technology for green fertilization and sustainable agricultural development under global carbon neutrality and green development goals (Li et al., 2021). Previous studies have shown that CFR+OA can significantly increase the content of soil labile carbon fractions, improve soil structure and nutrient release processes, and promote plant growth and quality improvement (Han et al., 2021; Zhang et al., 2024b; Xu et al., 2025). As vital exogenous carbon sources, organic amendments play a crucial role in regulating soil carbon cycling, promoting carbon sequestration, and improving soil fertility, thus facilitating crop growth and quality improvement (Chandel et al., 2024; Chen et al., 2024a; Zhang et al., 2024a; Lv et al., 2025). Different types of organic amendments have distinct characteristics in regulating soil: organic fertilizers enhance soil carbon stability and improve soil structure by providing abundant humus and nutrient sources (Zhang et al., 2022); bio-organic fertilizers enhance soil biological activity and nutrient conversion efficiency via functional microorganisms (Jin et al., 2022); biochar improves water retention, nutrient retention, and aeration owing to its high carbon stability and porous structure, offering long-term carbon sequestration potential (Liu et al., 2024a); vermicompost significantly increases soil enzyme activity and microbial community diversity by providing abundant active substances (Oyege and Balaji Bhaskar, 2023). For example, a 20% reduction in chemical fertilizers combined with organic fertilizer significantly increased, wheat yield, protein yield, and nitrogen use efficiency, while reducing nitrate nitrogen residue in the soil (Zhang et al., 2024b); an appropriate proportion of organic fertilizer replacement significantly improved maize yield and quality, increased SOC and its soil labile carbon fractions, the content of most secondary and micronutrients, and the carbon pool management index (CPMI), while minimizing heavy metal contamination risks (He et al., 2022a). However, current research on CFR+OA primarily focuses on field crops, with limited studies on high-value medicinal plants.

Currently, fertilization techniques for *A. membranaceus* cultivation are relatively outdated, characterized by inefficient nutrient management, excessive chemical fertilizer use, and insufficient organic inputs. These problems not only lead to a decline in both the yield and quality of *A. membranaceus* but also cause a reduction in soil organic matter content, posing environmental risks and severely restricting the sustainable development of the *A. membranaceus* industry (Wang et al., 2020; Hao et al., 2024; Zhang et al., 2025). Previous studies have confirmed that soil organic matter is a key factor restricting the yield and quality of *A. membranaceus*, yet its specific regulatory mechanism remains unclear (Sun et al., 2020). Therefore, achieving a synergistic improvement in yield and quality through scientific fertilization has become an urgent problem in *A. membranaceus* cultivation.

To address these issues, this study focuses on Hengshan *A. membranaceus* and aims to achieve the following objectives through field experiments: (i) evaluate the effects of different fertilization regimes on yield, quality, soil properties, and economic benefits, thereby clarifying the practical viability of CFR+OA technology in *A. membranaceus* cultivation; (ii) beyond basic soil physicochemical properties, further investigate soil labile carbon fractions—the most active components of soil organic matter—as well as soil enzyme activities, and elucidate the mechanisms behind the formation of *A. membranaceus* yield and quality under CFR+OA technology through an integrated multi-index analysis. Based on this, we hypothesize that CFR+OA technology can drive synergistic improvements in *A. membranaceus* yield and quality by enhancing the soil labile carbon pool. This study will provide a theoretical foundation and technical support to improve soil quality in production areas and to develop fertilization technology for *A. membranaceus* cultivation.

## 2 Materials and methods

This study builds upon previous research. In 2023, a bio-organic fertilizer efficiency trial conducted at two sites demonstrated that CFR+BOF effectively increased the yield and quality of *A. membranaceus* (**Supplementary Tables 1**, **2**, **Supplementary Figures 2**, **3**). This study aims to further evaluate the efficacy of bio-organic fertilizer, with a particular focus on elucidating the underlying mechanisms driving yield and quality enhancement. To identify the critical factors influencing the growth and quality formation of *A. membranaceus*, the experiment included treatments of organic fertilizer (without microbial inoculants), bio-organic fertilizer, and bioactive vermicompost. Precipitation and temperature characteristics for the 2023–2024 period are shown in **Supplementary Figures 1**, **S4**.

### 2.1 Materials and experimental site description

The experiment was conducted from April to October 2024 in Xiamayu Township, Ying County, Shuozhou City, Shanxi Province, China (113.13°E, 39.36°N). This area is located in the southern part of the Hengshan mountainous region, a major production region for Hengshan *A. membranaceus*. The study area has a temperate continental climate, with an average annual temperature of approximately 7-9 °C and an average annual precipitation of about 400–450 mm, which is mainly concentrated in July and August. The soil type was Calciustoll (light chestnut soil) with a sandy loam, and the preceding crop was spring maize. The properties of the 0–20 cm topsoil layer were as follows: bulk density (BD) of 1.52 g cm^-3^, pH of 8.35, soil organic matter (SOM) of 11.35 g kg^-1^, total nitrogen (TN) of 0.88 g kg^-1^, available phosphorus (AP) of 6.63 mg kg^-1^, and available potassium (AK) of 121.59 mg kg^-1^.

### 2.2 Experimental design

The experiment was arranged in a randomized complete block design with five fertilization treatments: farmer practice (FP), chemical fertilizer reduction (CFR), and three CFR+OA combinations, namely organic fertilizer (OF), bio-organic fertilizer (BOF), and vermicompost (Vm). Each treatment had three replications, with each plot measuring 63 m² (7 m × 9 m). Both chemical fertilizer and bio-organic fertilizer were independently developed by our research group. The organic fertilizer was prepared from cow manure. The bio-organic fertilizer was prepared using organic fertilizer as a carrier, with *Bacillus velezensis* C44 as the functional strain. To ensure comparability, nutrient inputs were kept consistent across CFR+OA treatments. Fertilizer application rates were calculated based on the physicochemical properties of the organic amendments (**Supplementary Table 3**), and the detailed fertilization scheme is provided in **Supplementary Table 4**. Except for fertilization, all other field management practices were kept consistent across treatments.

One-year-old locally cultivated *A. membranaceus* seedlings of uniform size and healthy growth (sown in spring 2023, lifted in spring 2024) were selected for this study. On April 7, 2024, the seedlings were transplanted at a density of 15 plants m^-2^ (plant spacing 20 cm, row spacing 28 cm). This planting practice was consistent with local standard agronomic practices.

### 2.3 Sample collection

At harvest, soil samples were randomly collected from each plot at two depth layers (0–20 cm and 20–40 cm) to analyze soil basic physicochemical properties, labile carbon fractions, and enzyme activity. Additionally, *A. membranaceus* plant samples were collected from a randomly selected 2 m × 2 m quadrat within each plot to measure growth parameters and active constituent content (AC).

### 2.4 Determination indices and methods

#### 2.4.1 Growth parameters of *A. membranaceus*

At harvest, shoot and root samples of *A. membranaceus* were collected and stored in kraft paper bags. The samples were dried at 105 °C for 30 minutes, then further dried at 65 °C until a constant weight was achieved. Shoot dry weight (SDW) and RDW were determined using an electronic balance. MRL and MRD were measured to the nearest 0.1 cm and 0.01 mm with a ruler and a digital caliper, respectively.

#### 2.4.2 Active constituent content of *A. membranaceus*

Quantitative analysis of astragaloside IV, calycosin-7-glucoside, ononin, formononetin, and calycosin was performed using ultra-performance liquid chromatography-tandem mass spectrometry (UPLC-MS/MS), following the method previously established by our group (Sun et al., 2025). The structures of the five analyte compounds in *A. membranaceus* samples are shown in **Supplementary Figure 6**.

##### 2.4.2.1 Preparing standard solutions

Individual stock solutions were prepared by accurately weighing 0.1 mg of astragaloside IV, calycosin-7-glucoside, ononin, formononetin, and calycosin in 80% methanol to a final volume of 10 mL. A mixed standard stock solution was subsequently prepared by combining 1 mL of each stock solution (astragaloside IV, calycosin-7-glucoside, ononin, formononetin, and calycosin), and diluting the mixture to 10 mL with 80% methanol. All solutions were stored at 4 °C prior to analysis. Reference standards were purchased from Tanmo Quality Inspection Reference Material Center (China).

##### 2.4.2.2 Preparing sample solutions

Dried root samples of *A. membranaceus* were pulverized using a high-speed mill and passed through a 0.25 mm sieve. Approximately 1.0 g (accurately weighed to 1.0000 g) of the powder was placed in a 250 mL round-bottom flask, then mixed with 50 mL of 80% methanol containing 4% concentrated aqueous ammonia. The initial weight was recorded. After standing for 30 minutes, the mixture was heated under reflux for 1 hour. After cooling to room temperature, the weight loss was made up with 80% methanol containing 4% concentrated aqueous ammonia. The solution was thoroughly mixed and filtered. A 25 mL aliquot of the filtrate was evaporated to dryness. The residue was redissolved in 80% methanol and diluted to volume in a 5 mL volumetric flask. It was then filtered through a 0.22 µm nylon syringe filter before analysis.

##### 2.4.2.3 Chromatographic conditions

Chromatographic separation was performed on a Waters ACQUITY UPLC BEH C18 column (2.1 mm × 50 mm, 1.7 μm) maintained at 40 °C. The injection volume was 2 μL. The mobile phase was delivered at 0.4 mL min^-^¹ and consisted of two components: acetonitrile (HPLC-grade) as Solvent A and 0.1% (v/v) formic acid in water (analytical-grade formic acid) as Solvent B. The gradient program was as follows: 0–1 min (10% A), 1–2 min (10 %-15 % A), 2–3 min (15 % A), 3–8 min (15%-40% A), 8–9 min (40% A), 9–10 min (40%-90% A), 10–11 min (90% A), and 11-11.1 min (90%-10% A). The chromatograms of the five analytes are shown in **Supplementary Figure 7**.

##### 2.4.2.4 Mass spectrometric conditions

Electrospray ionization (ESI) was employed in simultaneous positive and negative ion modes using multiple reaction monitoring (MRM). The source parameters were set as follows: source voltage, 2 kV; capillary temperature, 500 °C; cone voltage, 30 V; and desolvation gas flow rate, 1000 L h^-1^. The specific mass spectrometric parameters for the five compounds are shown in **Supplementary Table 5**.

##### 2.4.2.5 Method validation

The developed UPLC-MS/MS method was validated. The intra-day and inter-day precision (RSD) for all analytes were below 5.0%, and the recoveries ranged from 97.24% to 102.90%. These results indicated that the established method was accurate and reliable, and suitable for the quantitative determination of target analytes in this study. The comprehensive validation data, including linear regression equations, limits of detection (LOD), limits of quantification (LOQ), precision, and accuracy, are provided in **Supplementary Tables 6**, **7**.

#### 2.4.3 Soil basic physicochemical properties

Soil pH was determined using a pH meter in a 1:5 (w/v) soil-water suspension. SOM was determined using the potassium dichromate oxidation-external heating method. TN was measured by the Kjeldahl digestion method. Soil ammonium nitrogen (NH_4_^+^-N) and nitrate nitrogen (NO_3_^--^N) were extracted with KCl and analyzed using flow injection analysis. AP was determined using the Olsen method. AK was extracted with ammonium acetate and measured by flame photometry. All soil basic physicochemical properties were analyzed according to standard procedures described by Bao (2000) (Bao, 2000).

#### 2.4.4 Soil labile carbon fractions

POC was determined using the potassium dichromate oxidation method following wet sieving through a 53 µm sieve (Cambardella and Elliott, 1992). ROC was analyzed by the potassium permanganate oxidation method (333 mmol L^-^¹ KMnO_4_) (Blair et al., 1995). DOC was extracted with distilled water (1:5 w/v) (Jones and Willett, 2006) and measured using a Multi N/C 3100 analyzer (Analytik Jena, Germany). MBC was determined by the chloroform fumigation–potassium sulfate extraction method (Vance et al., 1987).

#### 2.4.5 Soil enzyme activities

Soil urease (S-UE) activity was determined by the sodium phenolate–sodium hypochlorite colorimetric method. Soil sucrase (S-SC) activity was assayed with the 3,5-dinitrosalicylic acid colorimetric method. Soil alkaline phosphatase (S-ALP) activity was measured using the disodium phenyl phosphate colorimetric method. All soil enzyme activities were determined using kits (Beijing Solarbio Science & Technology Co., Ltd.) according to the manufacturer’s instructions.

### 2.5 Data analysis methods

#### 2.5.1 Calculation of active constituent yield

This study introduced the ACY index to systematically evaluate the comprehensive effects of organic amendments on the medicinal value of *A. membranaceus*. The formula is as follows:


ACY=AC×RDW

where ACY (kg m^-2^) is the active constituent yield, AC (%, which represents the mass percentage of active constituents in the dried root powder) is the active constituent content, and RDW (kg m^-2^) is the root dry weight.

#### 2.5.2 Calculation of soil carbon pool management index

CPMI reflects the sensitive changes in soil organic carbon quality. The formula is as follows:


$$\text{CPI}=\text{SOC}/{\text{SOC}}_{0}$$

NLC=SOC−AOC

L=AOC/NLC

$$\text{LI}=\text{L}/{\text{L}}_{0}$$

CPMI=CPI×LI×100

where CPI is the carbon pool index, SOC (g kg^-1^) is the soil organic carbon, SOC_0_ (g kg^-1^) is the baseline SOC value from CFR treatment, AOC (g kg^-1^) is the active organic carbon (calculated using 333 mmol L^-1^ readily oxidizable organic carbon in this study), NLC (g kg^-1^) is the non-labile carbon (calculated as the difference between SOC and AOC), L is the carbon pool lability, LI is the lability index, L_0_ is the baseline carbon pool lability value from CFR treatment, and CPMI (%) is the carbon pool management index.

#### 2.5.3 Calculation of Fuzzy membership function

A fuzzy membership function was employed to normalize experimental data for a comprehensive evaluation of multiple indicators. The formulas are as follows:


$$X(f_{1})=(X-X_{min})/(X_{max}-X_{min})$$

$$X(f_{2})=(X_{max}-X)/(X_{max}-X_{min})$$

where *X*(*f*_1_) and *X*(*f*_2_) represent the fuzzy membership values for positively and negatively correlated indicators, respectively. *X* denotes the raw measured value of a specific indicator in *A. membranaceus*. The maximum and minimum values of the indicator across all samples are represented by *X_max_* and *X_min_*. *X*(*f*_1_) is used for positive indicators and *X*(*f*_2_) for negative indicators.

#### 2.5.4 Data processing and analysis methods

The chemical structures of *A. membranaceus* compounds were drawn using ChemDraw 22.0.0 (PerkinElmer Informatics, Waltham, MA, USA). Statistical analyses were performed in the R environment (R Core Team, Vienna, Austria; version 4.4.1). One-way ANOVA was used to test treatment effects, followed by multiple comparisons with the Least Significant Difference (LSD) method. Chromatograms and bar charts were generated using the plotly and ggplot2 packages, respectively. Mantel tests, Random Forest analysis, and linear regression analysis were constructed using the linkET package (GitHub - Hy4m/linkET: Everything is linkable, n.d.), rfPermute package (EricArcher/rfPermute: Version 2.5.4, n.d.), and stats package, respectively. Line plots and radar charts were created using Origin 2021 (OriginLab Corporation, Northampton, MA, USA). Finally, all figures were assembled and formatted using Microsoft PowerPoint 2024 to ensure a coordinated and professional layout.

## 3 Results

### 3.1 Effect of chemical fertilizer reduction combined with organic amendments on the yield and quality of *A. membranaceus*

**Figure 1:**
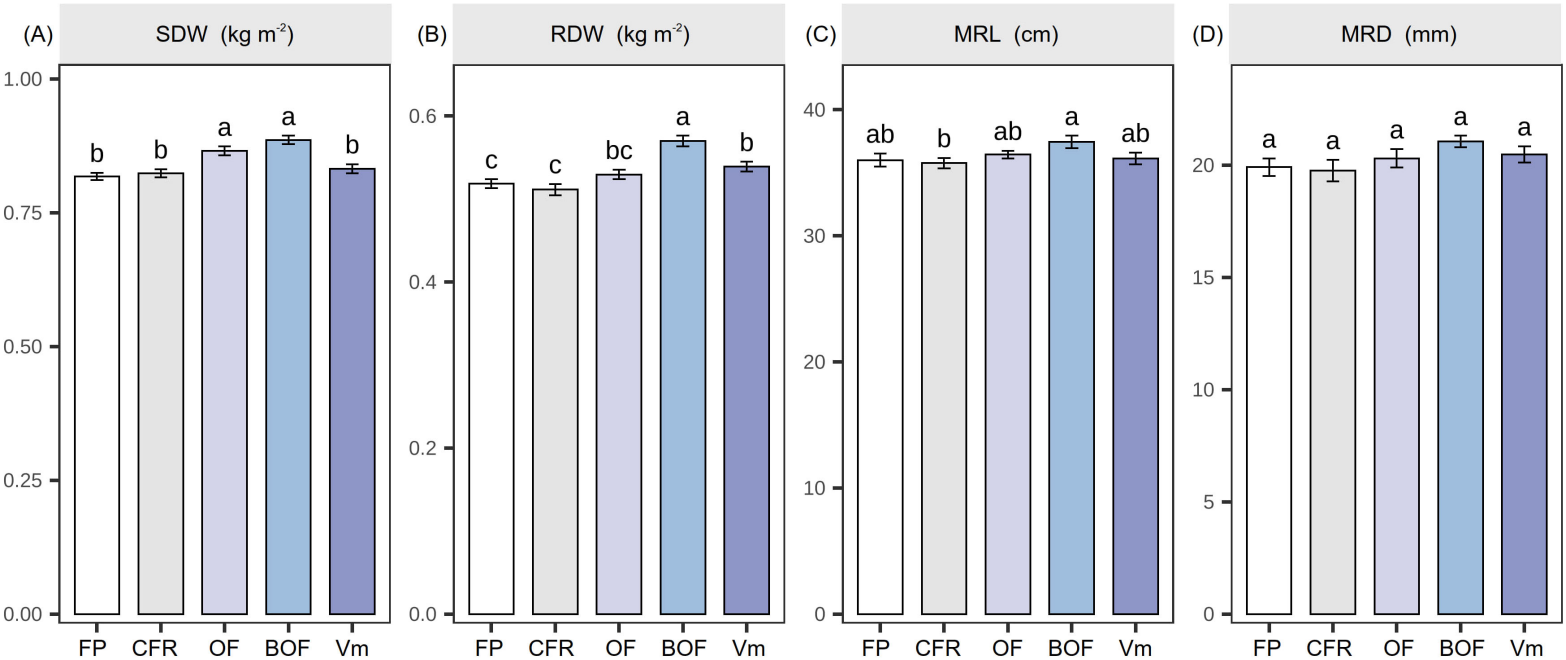
Effect of chemical fertilizer reduction combined with organic amendments on the growth indicators of *A. membranaceus*. **(A)** shoot dry weight (SDW); **(B)** root dry weight (RDW); **(C)** main root length (MRL); **(D)** main root diameter (MRD). The values are presented as the mean ± standard error. Different lowercase letters indicate significant differences between treatments at harvest (*P* <  0.05). Farmer practice (FP); chemical fertilizer reduction (CFR); chemical fertilizer reduction combined with organic fertilizer (OF); chemical fertilizer reduction combined with bio-organic fertilizer (BOF); chemical fertilizer reduction combined with vermicompost (Vm).

As shown in **Figure 1**, CFR+OA treatments enhanced biomass accumulation and root growth in *A. membranaceus*. SDW followed the order of BOF > OF > Vm > CFR > FP, and RDW followed the trend of BOF > Vm > OF > FP > CFR. BOF treatment showed the highest biomass. Compared with FP, BOF treatment increased SDW and RDW by 8.31% and 9.86%, respectively. CFR+OA treatments enhanced MRL and MRD, but no significant differences were observed among treatments.

**Figure 2:**
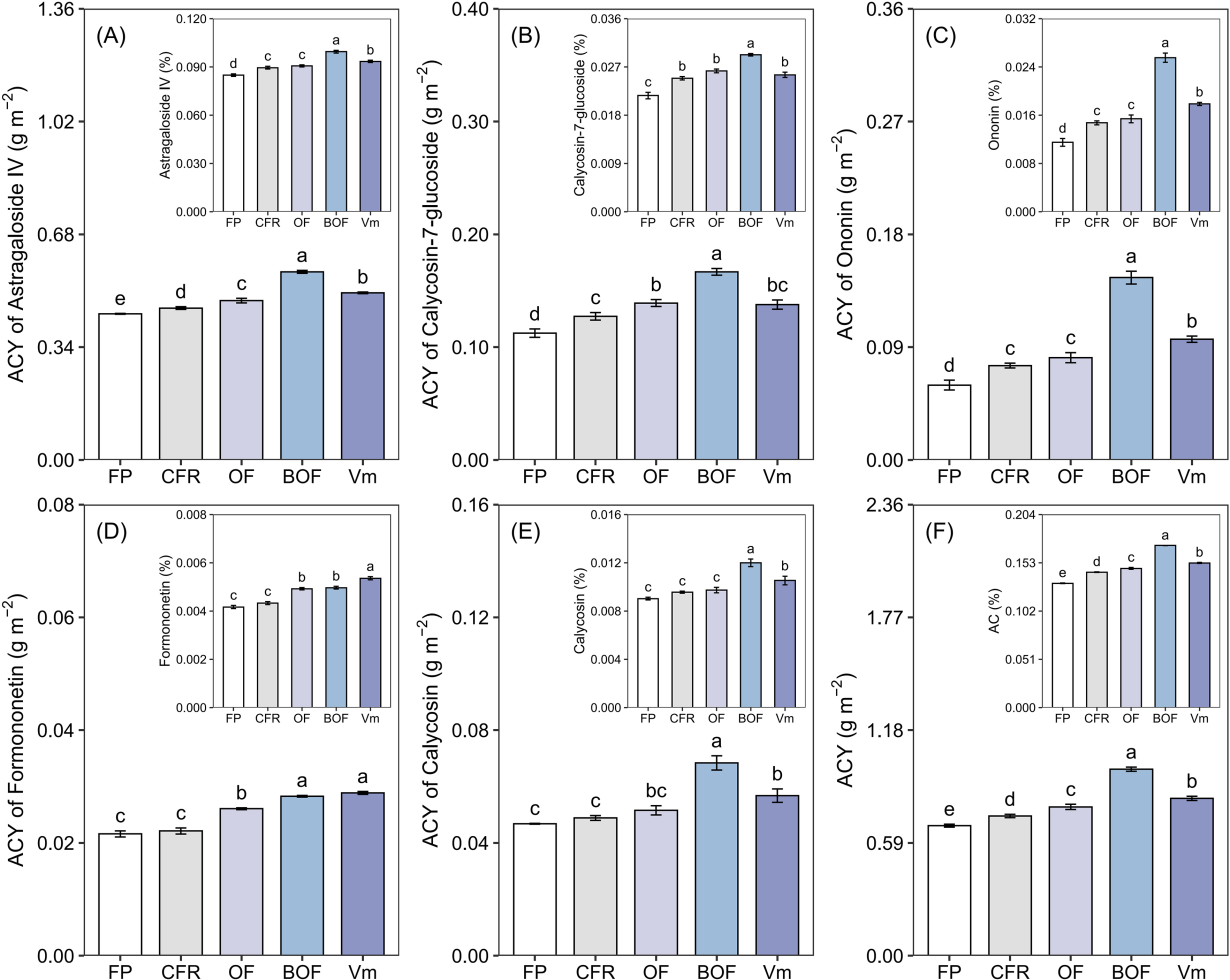
Effect of chemical fertilizer reduction combined with organic amendments on the active constituent yield of *A. membranaceus*. **(A)** ACY of Astragaloside IV; **(B)** ACY of Calycosin-7-glucoside; **(C)** ACY of Ononin; **(D)** ACY of Formononetin; **(E)** ACY of Calycosin; **(F)** ACY (sum of A–E). The main figure shows active constituent yield (ACY, g m^-^²). The subfigure shows the active constituent content (AC, %), which represents the mass percentage of active constituents in the dried root powder. The values are presented as the mean ± standard error. Different lowercase letters indicate significant differences between treatments at harvest (*P*  <  0.05). Farmer practice (FP); chemical fertilizer reduction (CFR); chemical fertilizer reduction combined with organic fertilizer (OF); chemical fertilizer reduction combined with bio-organic fertilizer (BOF); chemical fertilizer reduction combined with vermicompost (Vm).

This study used active constituent yield (ACY) to comprehensively evaluate the medicinal value of *A. membranaceus*. under the application of CFR+OA. As shown in **Figure 2**, CFR+OA treatments significantly promoted the accumulation of secondary metabolites in *A. membranaceus*. The ACY of astragaloside IV, ACY of calycosin-7-glucoside, ACY of ononin, and ACY of calycosin across different treatments all exhibited a trend of BOF > OF, Vm > CFR > FP. The BOF treatment demonstrated the most significant enhancement effect; compared with FP, ACY of astragaloside IV, ACY of calycosin-7-glucoside, and ACY of calycosin, increased by 28.71%, 48.39%, and 46.11%, respectively. The increase in ACY of ononin was particularly remarkable, reaching 143.70%. The overall effects of Vm and OF treatments followed, showing increases ranging from 9.02% to 61.35% compared with FP. ACY of formononetin exhibited a trend of Vm > BOF > OF > CFR > FP. Compared with FP, the Vm, BOF, and OF treatments increased ACY of formononetin by 33.62%, 30.88%, and 20.62%, respectively. Total ACY followed the trend of BOF > Vm > OF > CFR > FP. Compared with FP, the BOF, Vm, and OF treatments increased ACY by 43.31%, 20.94%, and 14.33%, respectively.

### 3.2 Effect of chemical fertilizer reduction combined with organic amendments on soil basic physicochemical properties

As shown in **Table 1**, after CFR+OA, the basic physical and chemical properties of the soil changed to varying degrees. Compared with FP, most soil properties showed an overall increasing trend, except for pH. The pH in the 0–20 cm and 20–40 cm layers decreased by 1.12%-2.12% and 0.04%-0.48%, respectively. The content of major nutrients, such as SOM, TN, AP, and AK, all increased. Specifically, SOM content increased by 20.28%-30.01% in the 0–20 cm layer and by 38.24%-52.52% in the 20–40 cm layer; TN content increased by 4.69%-28.12% in the 0–20 cm layer and by 1.08%-41.62% in the 20–40 cm layer; AP content increased by 37.97%-67.09% in the 0–20 cm layer and by 56.67%-83.33% in the 20–40 cm layer; AK content increased by 8.28%-17.79% in the 0–20 cm layer and by 9.35%-15.52% in the 20–40 cm layer. Different forms of nitrogen showed variation. NH_4_^+^-N content increased by 3.79% only in the BOF treatment in the 0–20 cm layer, while it decreased by 11.23% and 23.24% in the OF and Vm treatments, respectively. However, in the 20–40 cm layer, NH_4_^+^-N content increased by 39.20%-67.34%. NO_3_^--^N content in the 0–20 cm layer increased by 0.29% only in the BOF treatment, while those under OF and Vm treatments decreased by 17.52% and 3.02%, respectively; in the 20–40 cm layer, NO_3_^--^N content decreased by 34.36%-61.87%.

**Table 1:** Effect of chemical fertilizer reduction combined with organic amendments on the soil basic physicochemical properties.

Treatment	layer	pH	SOM	TN	NH_4_^+^-N	NO_3_^--^N	AP	AK
			(g kg^-1^)	(g kg^-1^)	(mg kg^-1^)	(mg kg^-1^)	(mg kg^-1^)	(mg kg^-1^)
FP	0–20 cm	8.35 ± 0.00a	9.55 ± 0.17d	0.85 ± 0.00c	2.64 ± 0.03a	4.28 ± 0.03a	5.27 ± 0.07d	135.18 ± 0.47d
	20–40 cm	8.33 ± 0.01A	5.83 ± 0.23C	0.62 ± 0.02B	0.41 ± 0.02D	5.59 ± 0.05A	3.00 ± 0.12C	114.46 ± 0.48C
CFR	0–20 cm	8.29 ± 0.01b	10.56 ± 0.17c	0.88 ± 0.01bc	2.27 ± 0.04bc	4.16 ± 0.02a	6.60 ± 0.12c	144.15 ± 0.59c
	20–40 cm	8.32 ± 0.01A	7.83 ± 0.04B	0.61 ± 0.01B	0.61 ± 0.02BC	3.79 ± 0.10B	4.67 ± 0.07B	121.70 ± 0.70B
OF	0–20 cm	8.21 ± 0.01c	11.88 ± 0.17ab	0.90 ± 0.01b	2.34 ± 0.04b	3.53 ± 0.02b	7.63 ± 0.09b	146.37 ± 0.49bc
	20–40 cm	8.32 ± 0.01A	8.14 ± 0.11B	0.84 ± 0.02A	0.69 ± 0.01A	3.67 ± 0.07B	4.70 ± 0.00B	125.23 ± 1.05B
BOF	0–20 cm	8.17 ± 0.01d	12.42 ± 0.15a	1.09 ± 0.01a	2.74 ± 0.07a	4.29 ± 0.06a	8.80 ± 0.12a	159.23 ± 0.59a
	20–40 cm	8.29 ± 0.01A	8.89 ± 0.11A	0.87 ± 0.01A	0.58 ± 0.02C	2.13 ± 0.04D	5.00 ± 0.12B	132.23 ± 1.17A
Vm	0–20 cm	8.25 ± 0.01bc	11.49 ± 0.06b	0.89 ± 0.01bc	2.02 ± 0.05c	4.15 ± 0.03a	7.27 ± 0.09b	147.59 ± 0.74b
	20–40 cm	8.32 ± 0.01A	8.06 ± 0.03B	0.62 ± 0.01B	0.67 ± 0.01AB	2.89 ± 0.07C	5.50 ± 0.06A	125.16 ± 1.05B

The values are presented as the mean ± standard error. Different lowercase letters indicate significant differences (*P* < 0.05) among treatments in the 0~20 cm layer, while different uppercase letters indicate significant differences (*P* < 0.05) among treatments in the 20~40 cm layer. Farmer practice (FP); chemical fertilizer reduction (CFR); chemical fertilizer reduction combined with organic fertilizer (OF); chemical fertilizer reduction combined with bio-organic fertilizer (BOF); chemical fertilizer reduction combined with vermicompost (Vm); soil organic matter (SOM); soil total nitrogen (TN); soil ammonium nitrogen (NH_4_^+^-N); soil nitrate nitrogen (NO_3_^--^N); soil available phosphorus (AP); soil available potassium (AK).

### 3.3 Effect of chemical fertilizer reduction combined with organic amendments on soil labile carbon pool and soil enzyme activity

**Figure 3:**
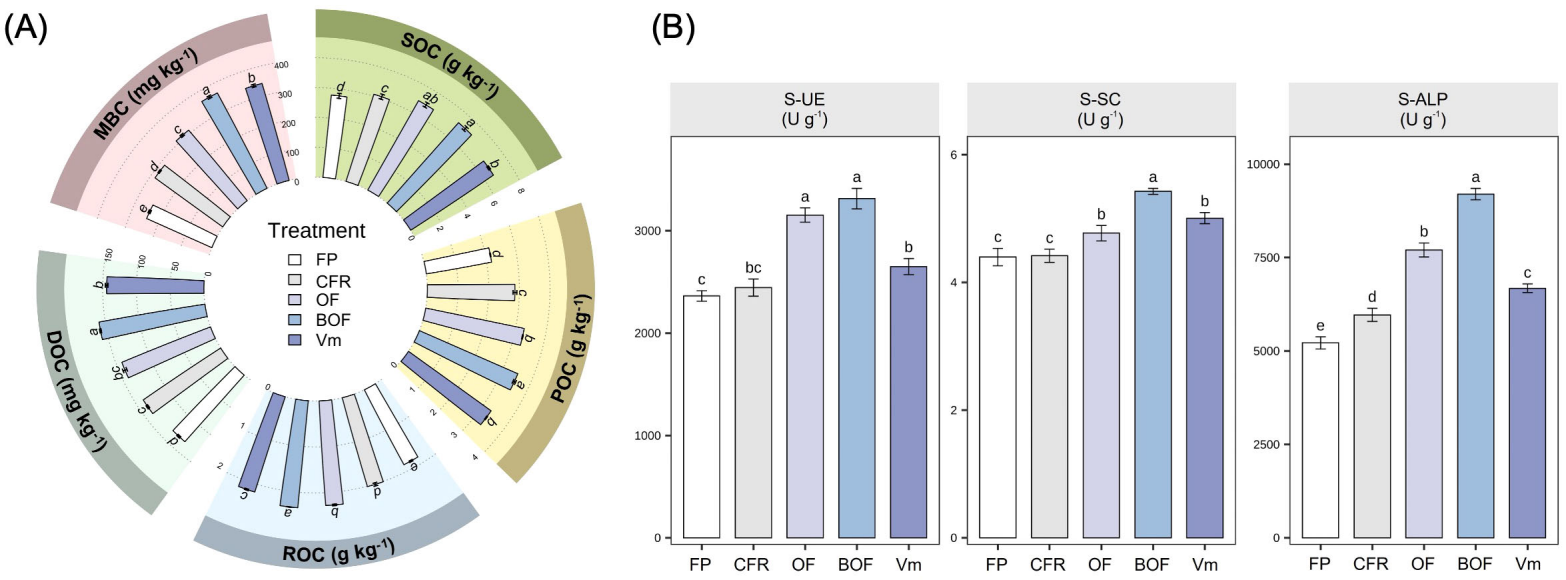
Effect of chemical fertilizer reduction combined with organic amendments on the soil labile carbon pool and soil enzyme activity. **(A)** soil labile carbon pool; **(B)** soil enzyme activity. The values are presented as the mean ± standard error. Different lowercase letters indicate significant differences between treatments at harvest (*P*  <  0.05). Farmer practice (FP); chemical fertilizer reduction (CFR); chemical fertilizer reduction combined with organic fertilizer (OF); chemical fertilizer reduction combined with bio-organic fertilizer (BOF); chemical fertilizer reduction combined with vermicompost (Vm); soil organic carbon (SOC); soil particulate organic carbon (POC); soil readily oxidizable organic carbon (ROC); soil dissolved organic carbon (DOC); soil microbial biomass carbon (MBC); soil urease (S-UE); soil sucrase (S-SC); soil alkaline phosphatase (S-ALP).

CFR+OA treatments significantly enhanced the soil carbon pool levels in the 0–20 cm soil layer (**Figure 3A**). The variation trends of soil organic carbon (SOC) and its labile fractions were basically consistent, exhibiting an overall trend of BOF > OF, Vm > CFR > FP. Compared with FP, the BOF, OF, and Vm treatments significantly increased SOC content by 30.01%, 24.31%, and 20.28%; POC content by 62.14%, 51.78%, and 53.59%; ROC content by 26.97%, 23.68%, and 18.09%; DOC content by 20.58%, 7.12%, and 9.19%; and MBC content by 50.50%, 28.27%, and 40.86%, respectively.

Soil enzyme activities exhibited variation trends similar to those of the labile carbon pool (**Figure 3B**). Compared with FP, CFR+OA treatments significantly enhanced the activities of S-UE, S-SC and S-ALP. Specifically, compared with FP, the BOF, OF, and Vm treatments significantly increased S-UE activity by 40.24%, 33.45%, and 12.14%; S-SC activity by 23.44%, 8.53%, and 13.86%; and S-ALP activity by 76.31%, 47.69%, and 27.98%, respectively.

### 3.4 Effects of chemical fertilizer reduction combined with organic amendments on the economic benefits of *A. membranaceus*

As shown in **Table 2**, compared with FP, CFR+OA treatments improved the economic benefits of *A. membranaceus* cultivation. The output value and net profit followed the order: BOF > Vm > OF > FP > CFR. Compared with FP, the net profits of the BOF, Vm, and OF treatments increased by 8.00%, 4.87%, and 3.10%, respectively. BOF generated the highest economic benefits, achieving a net profit of 25,603.04 USD ha^-1^, which was an increase of 1,897.23 USD ha^-1^ over the FP treatment.

**Table 2:** Effects of chemical fertilizer reduction combined with organic amendments on the economic benefits of *A. membranaceus*.

Treatment	Input cost (USD ha^-1^)			Yield (kg ha^-1^)	Output value (USD ha^-1^)	Net profit (USD ha^-1^)	Income increase (USD ha^-1^)
	Chemical fertilizer	Organic material	Additional cost (topdressing)				
FP	929.87	0.00	105.19	5188.31	24,740.88	23,705.81	0.00
CFR	315.57	0.00	0.00	5114.46	24,388.73	24,073.16	367.35
OF	315.57	504.91	0.00	5297.41	25,261.14	24,440.66	734.85
BOF	315.57	1262.27	0.00	5699.99	27,180.88	25,603.04	1,897.23
Vm	315.57	540.15	0.00	5392.70	25,715.52	24,859.80	1,153.99

Economic indicators were converted to USD at an exchange rate of 1 USD = 7.13 CNY. Farmer practice (FP); chemical fertilizer reduction (CFR); chemical fertilizer reduction combined with organic fertilizer (OF); chemical fertilizer reduction combined with bio-organic fertilizer (BOF); chemical fertilizer reduction combined with vermicompost (Vm). Chemical fertilizer cost includes both base fertilizer and topdressing fertilizer. Additional cost (topdressing) specifically refers to the additional labor required for the topdressing application. Other production costs (e.g., land preparation, transplanting, and harvesting) were consistent across all treatments and thus were not included in the calculation.

### 3.5 Comprehensive evaluation of different chemical fertilizer reduction combined with organic amendments

**Figure 4:**
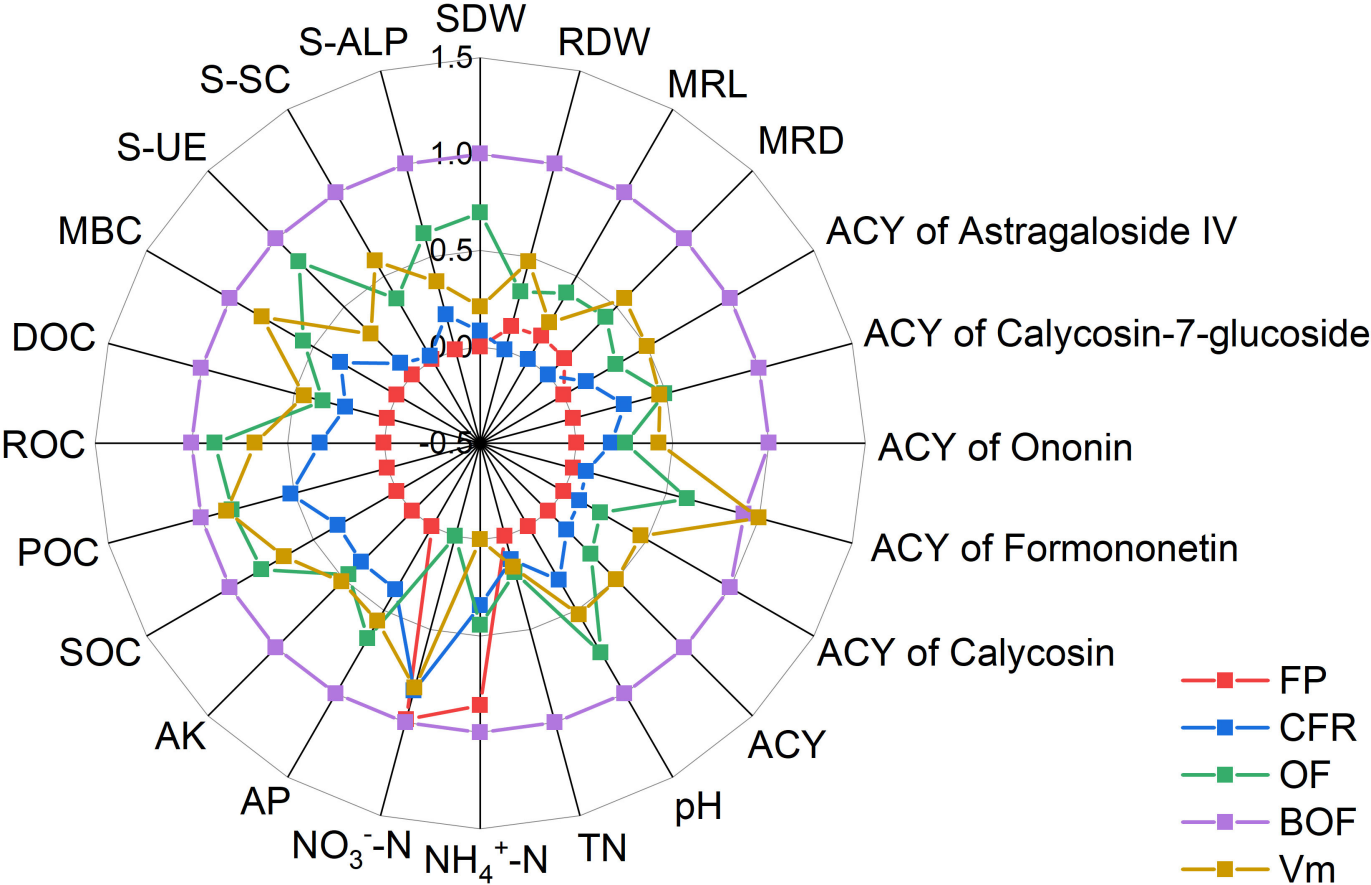
Fuzzy membership function analysis of influencing factors under chemical fertilizer reduction combined with organic amendments. Farmer practice (FP); chemical fertilizer reduction (CFR); chemical fertilizer reduction combined with organic fertilizer (OF); chemical fertilizer reduction combined with bio-organic fertilizer (BOF); chemical fertilizer reduction combined with vermicompost (Vm); shoot dry weight (SDW); root dry weight (RDW); main root length (MRL); and main root diameter (MRD); soil total nitrogen (TN); soil ammonium nitrogen (NH_4_^+^-N); soil nitrate nitrogen (NO_3_^--^N); soil available phosphorus (AP); soil available potassium (AK); soil organic carbon (SOC); soil particulate organic carbon (POC); soil readily oxidizable organic carbon (ROC); soil dissolved organic carbon (DOC); soil microbial biomass carbon (MBC); soil urease (S-UE); soil sucrase (S-SC); soil alkaline phosphatase (S-ALP); active constituent content (AC); active constituent yield (ACY).

The comprehensive evaluation based on fuzzy membership functions (**Figure 4**) ranked the treatments as follows: BOF > Vm > OF > CFR > FP. Among the CFR+OA treatments included in this study, BOF scored the highest (0.99), followed by Vm (0.55) and OF (0.36), all of which were considerably higher than FP (0.02).

### 3.6 Driving factors of yield and quality

**Figure 5:**
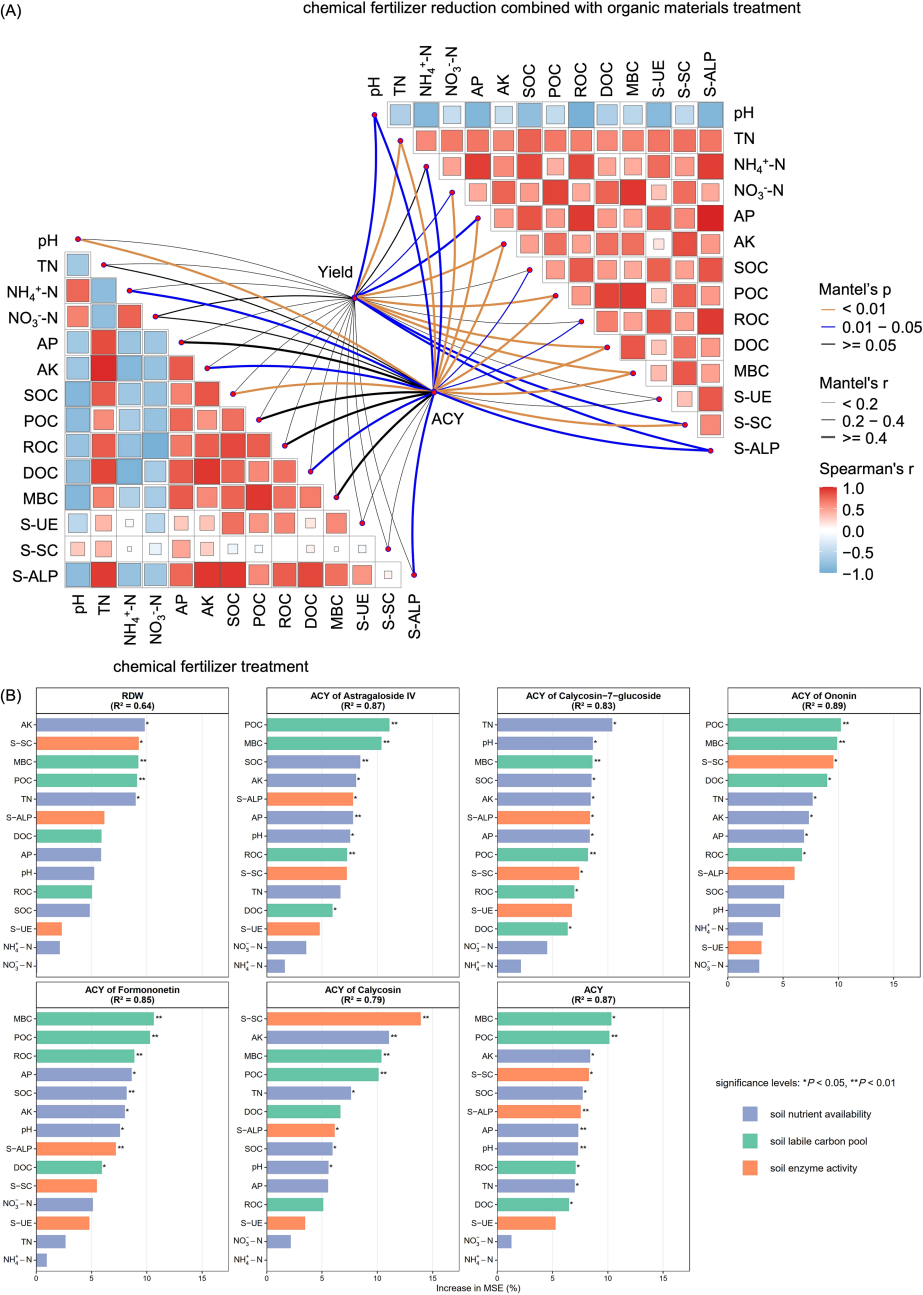
Multivariate statistical analysis linking soil properties to the yield and quality of *A. membranaceus*: **(A)** Mantel tests, **(B)** Random Forest analysis. **(A)** Mantel tests linking soil properties to Yield and ACY. The heatmap visualizes pairwise Spearman correlations between soil factors. Connecting lines denote Mantel test statistics, where line width corresponds to Mantel’s r value, and line color indicates the significance level (*P*-value). **(B)** Random Forest analysis results ranking the importance of individual soil predictors for Yield and ACY. The coefficient of determination (R^2^) indicates the proportion of variance explained by the model. Importance is quantified by the Increase in Mean Squared Error (%IncMSE). Significance levels: * *P* < 0.05 and ** *P* < 0.01. Soil total nitrogen (TN); soil ammonium nitrogen (NH_4_^+^-N); soil nitrate nitrogen (NO_3_^--^N); soil available phosphorus (AP); soil available potassium (AK); soil organic carbon (SOC); soil particulate organic carbon (POC); soil readily oxidizable organic carbon (ROC); soil dissolved organic carbon (DOC); soil microbial biomass carbon (MBC); soil urease (S-UE); soil sucrase (S-SC); soil alkaline phosphatase (S-ALP); active constituent yield (ACY).

Mantel test (**Figure 5A**) showed that the effects of soil basic physicochemical properties, labile carbon pool, and enzyme activities on RDW and ACY differed significantly among fertilization regimes. Under CFR+OA treatment, TN (r = 0.77), AK (r = 0.72), POC (r = 0.67), DOC (r = 0.68), and MBC (r = 0.56) showed extremely significant positive correlations with RDW (*P* < 0.01); all measured soil properties except S-UE were significantly correlated with ACY (*P* < 0.05). In contrast, under FP and CFR treatments, no significant correlations were observed between soil properties and RDW (*P* > 0.05), while only pH, NH_4_^+^-N, AK, SOC, DOC, and S-ALP were significantly correlated with ACY (*P* < 0.05). Spearman correlation analysis showed that under CFR+OA treatment, S-SC was strongly positively correlated with TN, AK, POC, DOC, and MBC (r > 0.80). In contrast, under FP and CFR treatments, S-SC showed weak or no correlation with all indices (|r| < 0.4).

**Figure 6:**
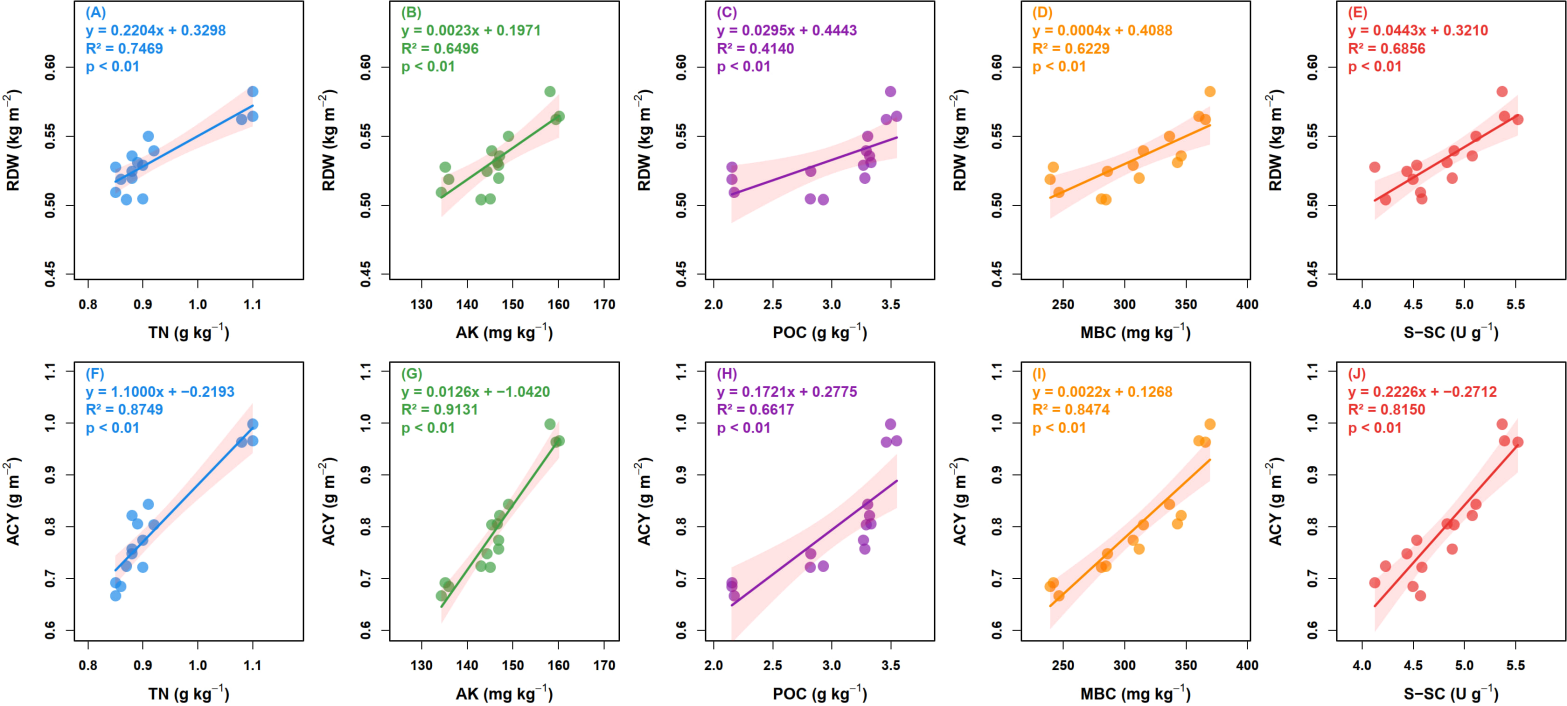
Linear regression analysis of key factors (TN, AK, POC, MBC, and S-SC) affecting the yield and quality of *A. membranaceus*. Root dry weight (RDW); active constituent yield (ACY); soil total nitrogen (TN); soil available potassium (AK); soil particulate organic carbon (POC); soil microbial biomass carbon (MBC); soil sucrase (S-SC).

Random Forest analysis (**Figure 5B**) identified TN, AK, POC, MBC, and S-SC as common key drivers (*P* < 0.05), collectively explaining the variation in yield (R² = 0.64) and quality (R² = 0.87) in *A. membranaceus*. Notably, among these five common factors, POC was the only one showing a highly significant positive correlation (*P* < 0.01) in both the yield and ACY models. Linear regression analysis (**Figure 6**) was performed to further quantify the impact of these key factors on RDW and ACY. The results demonstrated that RDW and ACY exhibited highly significant linear increases (*P* < 0.01) in response to rising levels of the identified soil properties.

In summary, CFR+OA technique influenced the yield and quality of *A. membranaceus* by improving soil physicochemical properties, labile carbon pools, and enzyme activities. TN, AK, POC, MBC, and S-SC were identified as the driving factors affecting the yield and quality. Among these, the soil labile carbon pool, dominated by POC, played an important role.

## 4 Discussion

### 4.1 Synergistic improvement of *A. membranaceus* yield, quality, and soil properties via chemical fertilizer reduction combined with organic amendments

*A. membranaceus* is a vital traditional Chinese medicinal crop whose cultivation aims not only to increase yields but, more importantly, to enhance the accumulation of active constituents. To evaluate the impact of different organic amendments on yield and quality and accurately quantify the medicinal value of *A. membranaceus*, this study adopted “active constituent yield (ACY)” as a core evaluation metric (Huang et al., 2024; Guo et al., 2025b). Compared with FP, CFR+OA treatments (OF, BOF, and Vm) significantly increased both the yield and ACY of *A. membranaceus* (**Figures 1**, **2**). This confirms the practical viability of CFR+OA technology. In terms of active constituent accumulation, organic amendment application significantly increased the content of flavonoids, specifically calycosin-7-glucoside, ononin, and formononetin. This is similar to existing conclusions. For instance, Guo et al. (2025a). reported that chemical fertilizer reduction combined with bio-organic or organic fertilizer significantly boosted flavonoid accumulation in *Scutellaria baicalensis* Similarly, Chandel et al. (2024) observed significantly increased eugenol content in *Ocimum gratissimum* L. following chemical fertilizer reduction combined with vermicompost. This improvement is probably because organic amendments can improve the rhizosphere environment and boost microbial activity. These changes subsequently upregulate the expression of key biosynthetic genes involved in secondary metabolism, thereby promoting the synthesis and accumulation of AC (Wang et al., 2022; Kumar et al., 2024).

Improving soil properties provides the material basis for enhancing crop yields and quality (He et al., 2018; Du et al., 2024; Xing et al., 2025). In maize production, applying organic-inorganic compound fertilizers increases soil organic matter, nutrient availability, and pH, thereby promoting crop growth and significantly enhancing grain yield and nutrient use efficiency (Chen et al., 2024b). Similarly, for wheat grown in saline-alkali soils, vermicompost ameliorated soil conditions by lowering electrical conductivity, bulk density, and penetration resistance, while boosting organic carbon, hydraulic conductivity, and aggregate stability, collectively creating an optimal soil environment for yield stability (Ding et al., 2021). Our findings corroborate these conclusions. Mantel test analysis revealed a high positive correlation among soil properties (excluding pH) under CFR+OA treatments, indicating that the input of organic amendments effectively enhanced soil fertility (**Figure 5A**). CFR+OA treatments not only significantly enhanced soil nutrient availability, the soil labile carbon pool, and enzyme activities (**Table 1**, **Figure 3**), but also played a crucial role in pH regulation. In this study, all indicators (excluding NO_3_^--^N) were negatively correlated with pH (**Figure 5A**), suggesting that the relatively high pH observed in FP treatment might limit *A. membranaceus* growth. This observation parallels the report by Hou et al. (2025b), who noted that amendments such as *Bacillus subtilis*, *Bacillus mucilaginosus*, humic acid, biochar, and vermicompost reduced soil pH to varying degrees in *Scutellaria baicalensis* cultivation. Since *A. membranaceus* prefers neutral to slightly alkaline conditions, adding organic amendments likely facilitated the release of organic acids and humus during decomposition. This process provided buffering and regulatory effects that lowered the initially high soil pH to an optimal level for root development, thereby creating a rhizosphere environment conducive to increased yield and quality (Adebanjo-Aina and Oludoye, 2025; Elumalai et al., 2025).

The BOF treatment demonstrated superior performance among the tested groups, achieving the highest score in the fuzzy membership function analysis (**Figure 4**). Functional microbes within the bio-organic fertilizer likely drive these benefits by secreting metabolites that stimulate root growth, optimize the rhizosphere environment, and accelerate soil nutrient cycling. Consequently, these mechanisms enhance nutrient uptake efficiency and stress resistance, thereby ensuring high-quality and high-yield *A. membranaceus* production (Huang et al., 2025). In conclusion, this study confirms that CFR+OA improves the soil environment, synergistically enhancing the yield and quality of *A. membranaceus*. Future research should integrate metabolomics and microbial community analysis to further elucidate the metabolic regulatory pathways and microbial mechanisms by which organic amendments enhance quality. This will provide a deeper understanding of high-quality *A. membranaceus* cultivation from a plant-soil interaction perspective.

### 4.2 Soil labile carbon fractions drive yield and quality improvement in *A. membranaceus*: POC as a key factor for high quality and high yield

The application of organic amendments enhances soil carbon stocks, preferentially driving the rapid accumulation and turnover of the labile carbon pool (Wei et al., 2025). In this study, CFR+OA treatments significantly boosted the contents of SOC and its soil labile carbon fractions (**Figure 3A**). These findings align with previous studies (Chen et al., 2024a; Wu et al., 2024). Further analysis of the ratio of active organic carbon (AOC) to SOC revealed that the increase in the POC/SOC ratio was most pronounced following the application of organic amendment (**Supplementary Figure 8**). This identifies POC as the labile fraction most sensitive to organic amendments. POC serves as a vital labile carbon fraction distinguished by its high bioavailability and nutrient-supply capacity (Guo et al., 2024). It fosters soil aggregate formation and stabilization, thereby significantly boosting soil aeration, structural stability, and water-nutrient retention capabilities (Tian et al., 2022; Zhou et al., 2025). *A. membranaceus* is a typical deep-rooting medicinal plant that thrives in loose, well-aerated soils with high nutrient retention (Zhang et al., 2024c). Our results indicate that increasing POC content improved soil properties, thereby creating a favorable soil environment for the growth and development of *A. membranaceus*. Additionally, the CPMI comprehensively reflects the impact of management practices on carbon pool quality and lability (Dai et al., 2021a; Mir et al., 2023). In this study, CPMI values in the CFR+OA treatments were significantly higher than those in the FP treatment (**Supplementary Table 4**), indicating that the application of organic amendments enhanced SOC content and carbon pool activity. Notably, the increasing trend in CPMI was consistent with changes in the POC/SOC ratio (**Supplementary Table 8**, **Supplementary Figure 8**), whereas the ROC/SOC ratio remained stable, implying that POC activation and accumulation played a dominant role in improving the quantity and quality of the soil carbon pool. This result not only demonstrates that the accumulation of POC is a key determinant of SOC increase (Chen et al., 2024a), but also aligns with the perspective that soil organic carbon accumulation in Northern China is primarily governed by aggregate protection mechanisms (Ma et al., 2024).

Given its multifaceted regulation of soil nutrient availability, structural stability, and carbon persistence, POC acts as a pivotal link between the soil environment and crop growth (Witzgall et al., 2021). In this study, CFR+OA significantly increased POC content (**Figure 3A**), which was positively and significantly correlated with yield, quality, and major soil factors (excluding pH) (**Figure 5A**). In the yield model, AK exhibited the highest relative importance, likely due to its role in regulating root cell expansion and phloem transport, thereby promoting the accumulation of *A. membranaceus* root biomass (Sustr et al., 2019), while the ACY model showed that MBC and POC primarily regulated the accumulation of active constituents (**Figure 5B**). As the primary driving factor, MBC suggests that active rhizosphere microbial communities may induce the synthesis and accumulation of secondary metabolites by triggering plant signaling pathways (Pang et al., 2021). This finding indicates that the POC-dominated soil labile carbon pool is a key factor in synergistically enhancing the quality and yield of *A. membranaceus*. This is likely because POC acts as a labile carbon source, providing metabolic substrates for MBC and driving secondary metabolite accumulation; concurrently, it improves soil physical structure, thereby promoting root development and nutrient uptake. These results are similar to those of Sun et al. (2020) and Ling et al. (2025), further validating that enhancing soil quality through organic matter management is a critical pathway for safeguarding *A. membranaceus* yield and quality. Therefore, organic matter management should be prioritized in *A. membranaceus* cultivation. Targeting the soil labile carbon pool to enhance carbon cycling and nutrient activation can achieve the goals of “carbon-fertilizes-soil, carbon-boosts-yield”. This study provides novel insights into the regulatory mechanisms of soil labile carbon on medicinal plant growth, offering a scientific basis for fertilization and soil management of medicinal plants.

### 4.3 Chemical fertilizer reduction combined with bio-organic fertilizer is a practical and promising strategy: Achieving soil-improving, yield- and quality-enhancing, and cost-effective goals in *A. membranaceus*

CFR+OA is an important fertilization technique in agricultural production. Its advantages in improving the ecological environment and promoting stable, increased crop yields have been verified in diverse cropping systems, including lettuce, maize, and *Scutellaria baicalensis* (He et al., 2022a; Jin et al., 2022; Guo et al., 2025a). This study demonstrates that the technique is also significantly effective in *A. membranaceus* cultivation. In terms of nutrient inputs, the FP treatment applied pure N, P, and K at 234.6, 205.5, and 302.25 kg ha^-1^, respectively. In contrast, the CFR+OA treatments applied pure N, P, and K at 90, 112.5, and 60 kg ha^-1^, representing substantial reductions of 61.6%, 45.3%, and 80.1%, respectively, compared to FP (**Supplementary Table 4**). Regarding fertilization management, the FP treatment required multiple top-dressing applications of chemical fertilizer during the middle and late growth stages, which increased labor inputs and management costs. Conversely, the CFR+OA strategy used slow-release compound fertilizer, which gradually released nutrients to match the nutrient needs of *A. membranaceus*. This method allowed for single fertilization without top-dressing, which lowers management costs and labor input. Compared with FP, the OF, BOF, and Vm treatments increased net income by 3.10%, 8.00%, and 4.87%, respectively (**Table 2**). Evidently, the CFR+OA fertilization technique achieves cost savings and increased income for *A. membranaceus*, holding great potential for widespread application.

Among the various treatments, chemical fertilizer reduction combined with bio-organic fertilizer emerged as the superior strategy, achieving the highest cost-effectiveness (**Figure 4**, **Table 2**). This success is mainly attributed to the functional microbes contained in the bio-organic fertilizer. The bio-organic fertilizer used in this study was developed by our research group. Its core functional strain, *Bacillus velezensis* C44, was isolated from soil samples in the Hengshan *A. membranaceus* production area and screened as a superior strain with both growth-promoting and disease-resistance properties (**Supplementary Figure 5**), including phosphate solubilization, IAA production, and siderophore secretion (Ma, 2023). Accordingly, our group developed a microbial inoculant with this strain by optimizing the fermentation process and carrier formula, which was later used to produce the bio-organic fertilizer (Ma et al., 2023). The results of this study not only validate the practical value of C44 but also provide a foundation for the future development of high-efficiency compound microbial agents.

In summary, chemical fertilizer reduction combined with bio-organic fertilizer achieved multiple benefits as a soil-improving, yield- and quality-enhancing, and cost-effective strategy, offering great potential for application in *A. membranaceus* cultivation. Future research will employ techniques such as high-throughput sequencing of microbial communities to further unravel the “plant-soil-microbe” interaction mechanisms following bio-organic fertilizer application, thereby providing a more comprehensive understanding of its underlying mechanisms in *A. membranaceus* cultivation.

## 5 Conclusions

**Figure 7:**
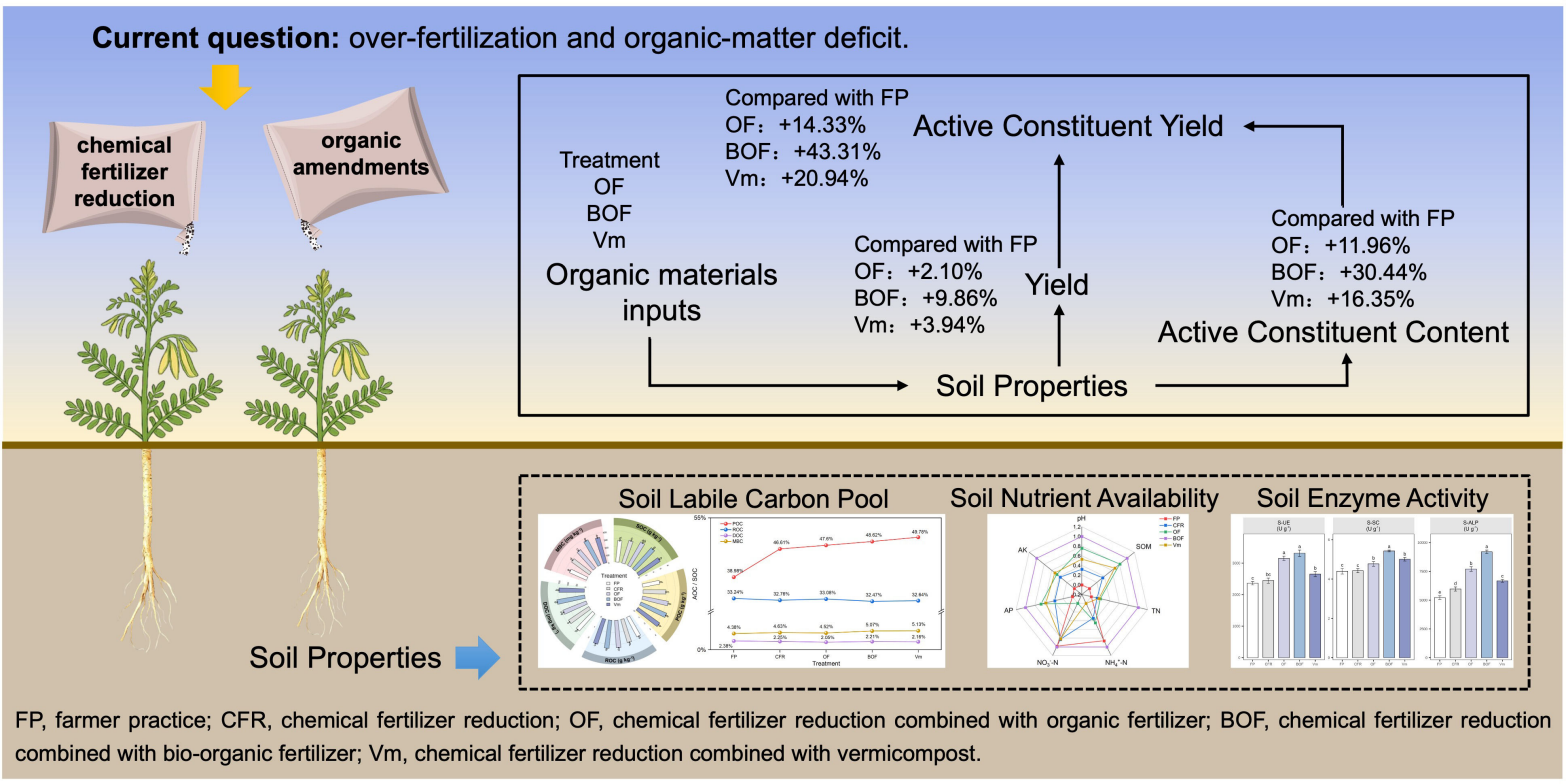
A conceptual framework illustrating the effects of chemical fertilizer reduction combined with different organic amendments on the yield, quality, and soil properties of *A. membranaceus.* The application of chemical fertilizer reduction combined with different organic amendments improves soil physicochemical properties, augments the soil labile carbon pool, and enhances soil enzyme activities, thereby synergistically improving yield and quality of *A. membranaceus*.

Addressing the challenges of over-fertilization and organic-matter deficit in the cultivation of the medicinal plant *A. membranaceus*, this study investigated the effects and regulatory mechanisms of CFR+OA technology on the growth and quality of *A. membranaceus* (**Figure 7**). The results indicate that CFR+OA technology achieved a synergistic improvement of yield and quality, significantly promoting the accumulation of key active constituents such as astragaloside IV and calycosin-7-glucoside. The CFR+OA technique significantly improved the soil environment and increased the contents of available soil nutrients (AP, AK), labile organic carbon fractions, and enzyme activities. Multivariate analysis demonstrated that TN, AK, POC, DOC, and MBC were important factors affecting the yield and quality of *A. membranaceus*, with the soil labile carbon pool, dominated by POC, being the key factor driving the high quality and high yield of *A. membranaceus*. Comparative analysis revealed that among different treatments, CFR+BOF (750 kg ha^-^¹ chemical fertilizer and 4500 kg ha^-^¹ bio-organic fertilizer) exhibited superior overall performance. Compared with FP, the yield and ACY of *A. membranaceus* under BOF treatment significantly increased by 9.86% and 43.31%, respectively, with an increase in economic benefits of 1,897.23 USD ha^-1^. Consequently, the application of CFR+BOF is recommended as an optimal fertilization strategy for the cultivation of *A. membranaceus*, providing a theoretical basis and practical support for green production of medicinal plants and improvement of soil fertility. Future research will employ omics technologies to further elucidate the targeted regulatory mechanisms by which bio-organic fertilizer improves the quality of *A. membranaceus*.

## Data Availability

The raw datasets presented in this article are not readily available because they are supporting ongoing studies. Requests to access the datasets should be directed to Junling Guo, sxauguojl@126.com.
