# Supplementary material for: Labile carbon drives synergistic improvements in Astragalus membranaceus yield and quality under chemical fertilizer reduction combined with organic amendments
Source: Front Plant Sci. 2026 May 1;17:1809778. doi: 10.3389/fpls.2026.1809778 (PMC13178292; doi:10.3389/fpls.2026.1809778)
Supplement: Supplementary file 1 [file DataSheet1.docx]

**Appendix A**

The appendix contains the meteorological data (Figure S1), fertilization rates (Table S1), growth indicators (Figure S2), quality indicators (Figure S3), and economic benefits (Table S2) of the 2023 bio-organic fertilizer trial.


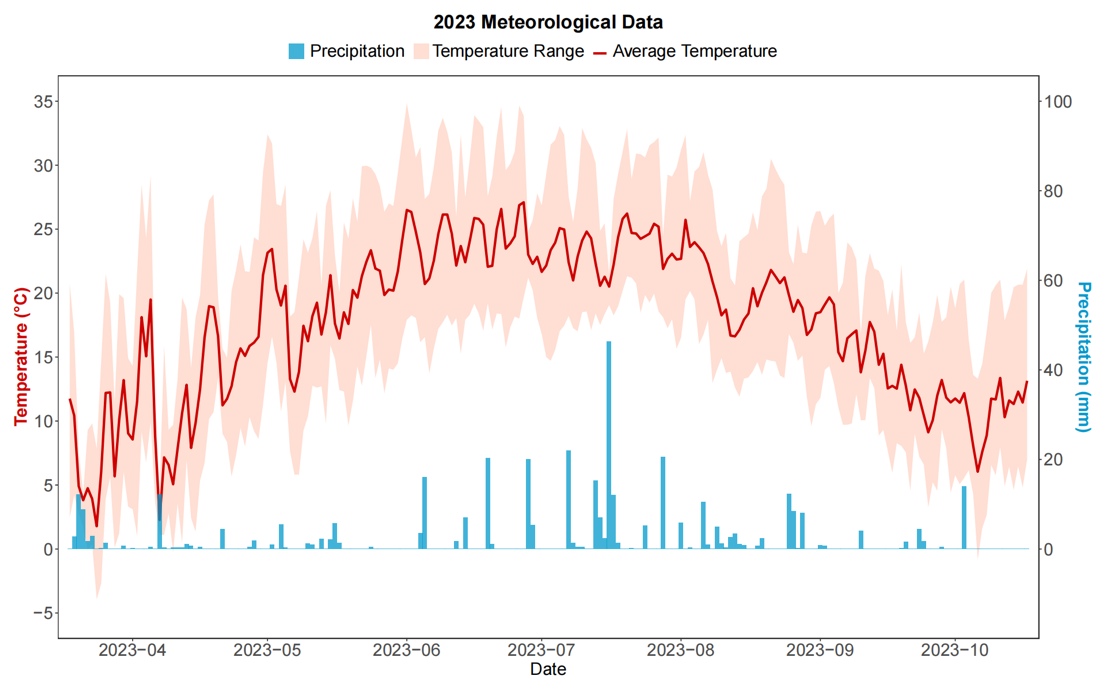


Figure S1. Meteorological data during the growth periods of 2023

Note: The shaded area represents the daily maximum and minimum temperature range; the red line denotes the daily mean temperature; the blue bars indicate the daily accumulated precipitation (Data source: ERA5 reanalysis).

Table S1. Evaluation of bio-organic fertilizer efficiency: fertilization rates of different treatments (2023)

| Treatment | Chemical fertilizer  (kg ha^-1^) | Chemical N  (kg ha^-1^) | Chemical P_2_O_5_  (kg ha^-1^) | Chemical K_2_O  (kg ha^-1^) | Organic material  (kg ha^-1^) |
| --- | --- | --- | --- | --- | --- |
| FP | 975(B)+600(T)+60(U) | 234.6 | 205.5 | 302.3 | 0 |
| OF | 750(SR) | 90.0 | 112.5 | 60.0 | 4500 (organic fertilizer) |
| BOF | 750(SR) | 90.0 | 112.5 | 60.0 | 4500 (bio-organic fertilizer) |

Note: Farmer practice (FP); chemical fertilizer reduction combined with organic fertilizer (OF); chemical fertilizer reduction combined with bio-organic fertilizer (BOF); B, base compound fertilizer (N-P₂O₅-K₂O: 12-18-15, total nutrients ≥ 45%); T, topdressing compound fertilizer (N-P₂O₅-K₂O: 15-5-26, total nutrients ≥ 46%); U, urea (N content 46%); SR, slow-release compound fertilizer (N-P₂O₅-K₂O: 12-15-8, total nutrients ≥ 35%).


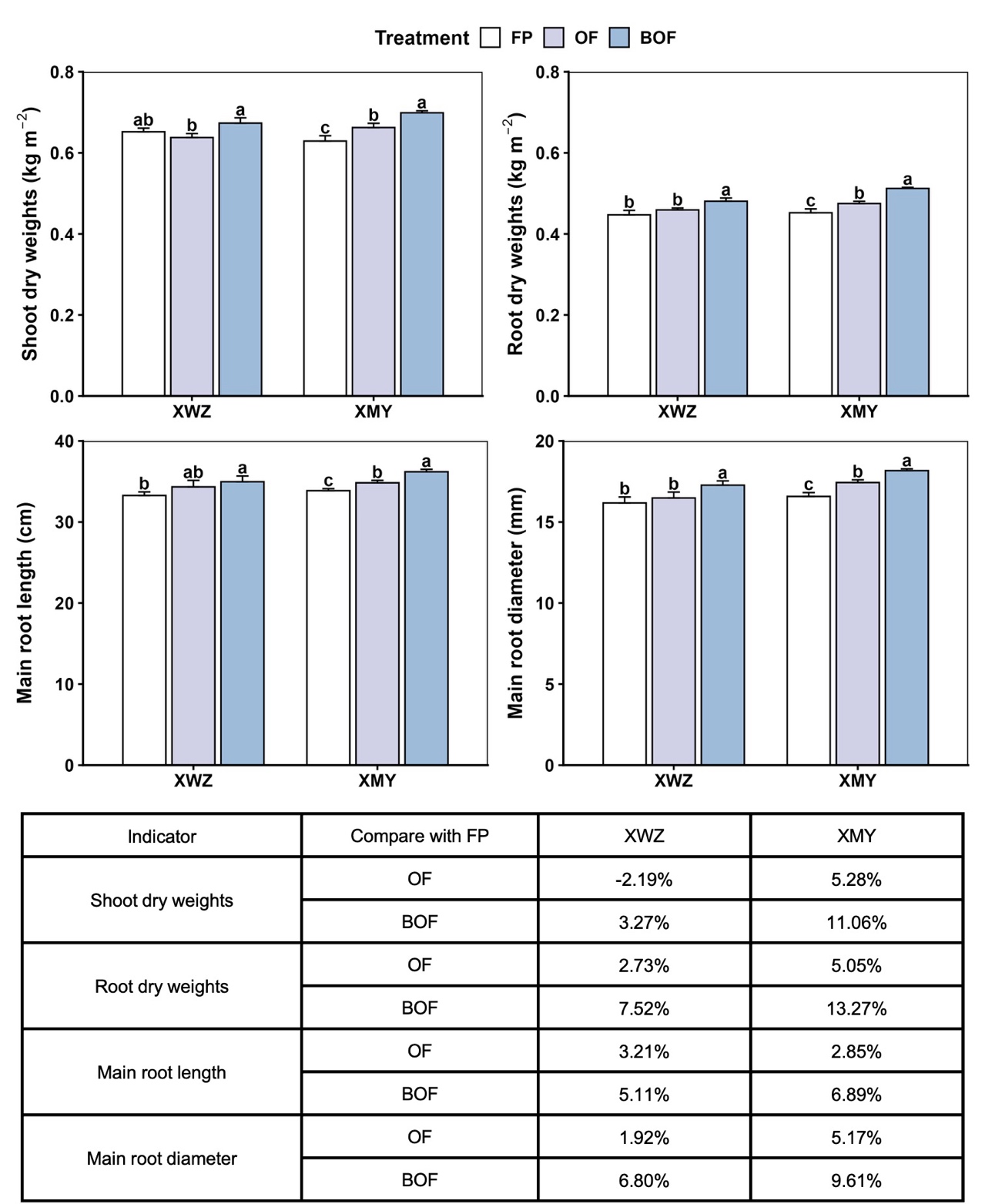


Figure S2. Evaluation of bio-organic fertilizer efficiency: growth indicators (2023)

Note: Different lowercase letters indicate significant differences between treatments within the same site (*P* <  0.05). XWZ (113.12°E, 39.47°N) and XMY (113.12°E, 39.40°N) are the two sites in Yingxian, Shanxi, China. The application rates for all treatments in the 2023 trials were the same as those used in this manuscript. FP, farmer practice; OF, chemical fertilizer reduction combined with organic fertilizer; BOF, chemical fertilizer reduction combined with bio-organic fertilizer.


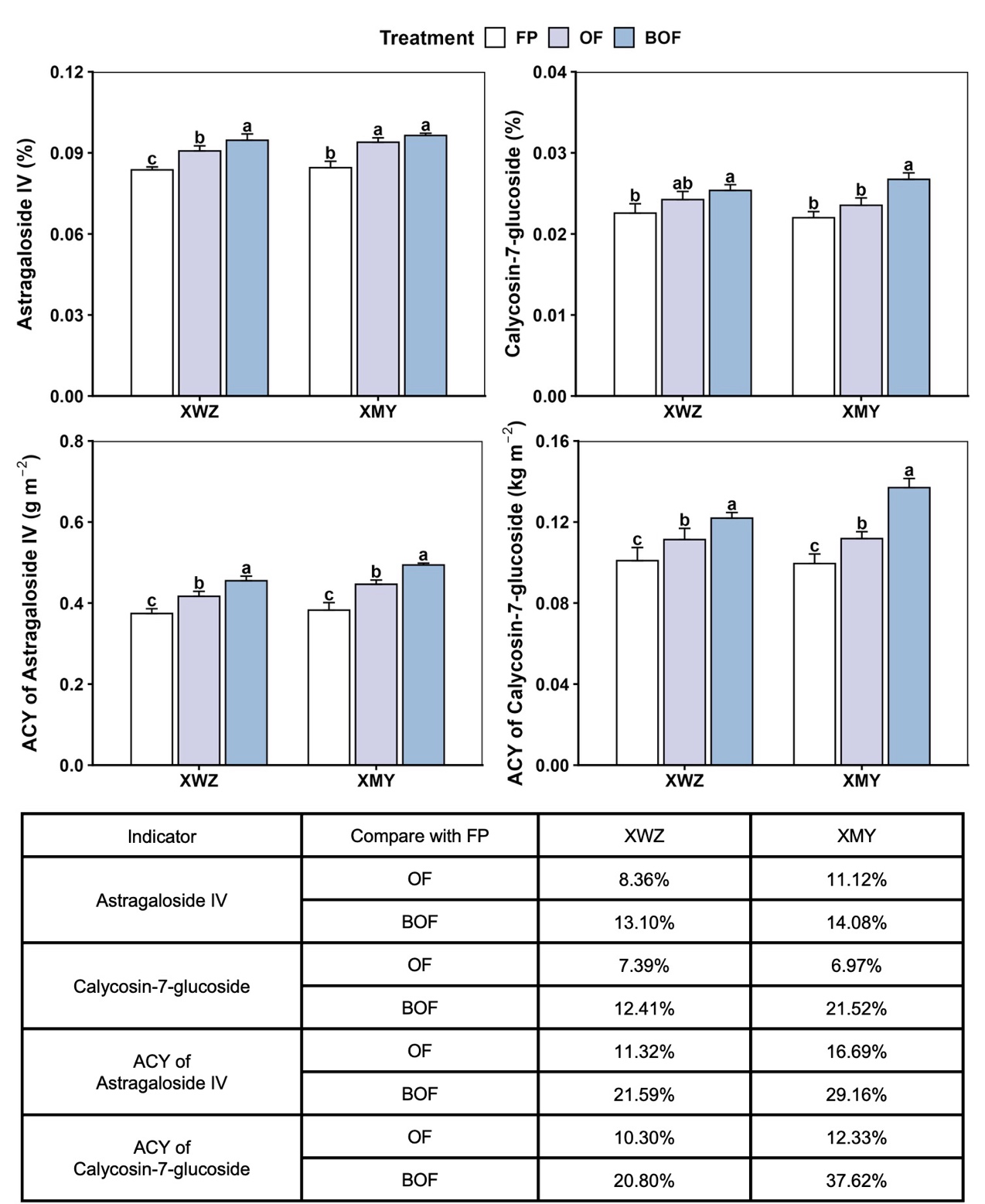


Figure S3. Evaluation of bio-organic fertilizer efficiency: quality indicators (2023)

Note: Different lowercase letters indicate significant differences between treatments within the same site (*P* <  0.05). XWZ (113.12°E, 39.47°N) and XMY (113.12°E, 39.40°N) are the two sites in Yingxian, Shanxi, China. The application rates for all treatments in the 2023 trials were the same as those used in this manuscript. FP, farmer practice; OF, chemical fertilizer reduction combined with organic fertilizer; BOF, chemical fertilizer reduction combined with bio-organic fertilizer.

Table S2. Evaluation of bio-organic fertilizer efficiency: economic benefits (2023)

| Sites | Treatment | Input cost (USD ha^-1^) | | | Yield  （kg ha^-1^） | Output  Value  （USD ha^-1^） | Net  Profit  （USD ha^-1^） | Income  Increase  （USD ha^-1^） |
| --- | --- | --- | --- | --- | --- | --- | --- | --- |
|  |  | Chemical  fertilizer | Organic  material | Additional cost  (topdressing) |  |  |  |  |
| XWZ | FP | 929.87 | 0.00 | 105.19 | 4472.71 | 21328.5 | 20293.43 | 0.00 |
|  | OF | 315.57 | 504.91 | 0.00 | 4594.68 | 21910.1 | 21089.62 | 796.19 |
|  | BOF | 315.57 | 1262.27 | 0.00 | 4809.07 | 22932.45 | 21354.61 | 1061.18 |
| XMY | FP | 929.87 | 0.00 | 105.19 | 4524.64 | 21576.13 | 20541.07 | 0.00 |
|  | OF | 315.57 | 504.91 | 0.00 | 4753.33 | 22666.64 | 21846.16 | 1305.09 |
|  | BOF | 315.57 | 1262.27 | 0.00 | 5070 | 24176.72 | 22598.88 | 2057.81 |

Note: Economic indicators were converted to USD at an exchange rate of 1 USD = 7.13 CNY. XWZ (113.12°E, 39.47°N) and XMY (113.12°E, 39.40°N) are the two sites in Yingxian, Shanxi, China. Farmer practice (FP); chemical fertilizer reduction combined with organic fertilizer (OF); chemical fertilizer reduction combined with bio-organic fertilizer (BOF). Chemical fertilizer cost includes both base fertilizer and topdressing fertilizer. Additional cost (topdressing) specifically refers to the additional labor required for the topdressing application. Other production costs (e.g., land preparation, transplanting, and harvesting) were consistent across all treatments and thus were not included in the calculation.

**Appendix B**

The appendix contains the meteorological data (Figure S4), organic amendment properties (Table S3), fertilization rates (Table S4), and bio-organic fertilizer specification (Figure S5) of the 2024 trial.


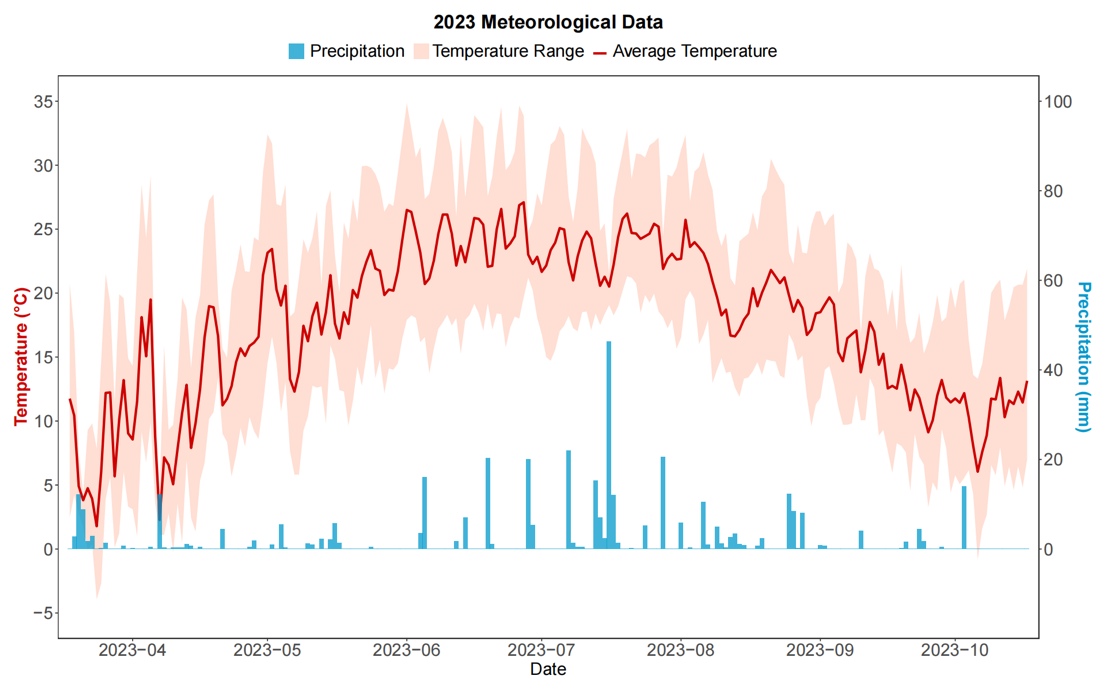


Figure S4. Meteorological data during the growth periods of 2024.

Note: The shaded area represents the daily maximum and minimum temperature range; the red line denotes the daily mean temperature; the blue bars indicate the daily accumulated precipitation (Data source: ERA5 reanalysis).

Table S3. Physicochemical and biological properties of the organic amendments used in this study (2024)

| Amendments | pH | Moisture content  (%) | OM | Total N | Total P_2_O_5_ | Total K_2_O | Effective viable count |
| --- | --- | --- | --- | --- | --- | --- | --- |
|  |  |  | (%) | (%) | (%) | (%) | (cfu g^-1^) |
| organic fertilizer | 8.20 | 34.07 | 34.43 | 1.78 | 1.65 | 2.57 | - |
| bio-organic fertilizer | 8.20 | 34.07 | 34.43 | 1.78 | 1.65 | 2.57 | 1.0×10⁹ |
| vermicompost | 8.30 | 53.29 | 53.60 | 1.84 | 2.30 | 1.60 | - |

Note: Organic matter (OM). The application rate of vermicompost (5,925 kg ha^-1^) was determined based on the equivalent total nutrient input principle, calculated in reference to the physicochemical properties (moisture content and dry-basis nutrient density) provided in Table S4. The bio-organic fertilizer used in this study was independently developed by our research group, and the functional microbial strain is *Bacillus velezensis* C44.

Table S4. Fertilization rates of different treatments (2024)

| Treatment | Chemical fertilizer  (kg ha^-1^) | Chemical N  (kg ha^-1^) | Chemical P_2_O_5_  (kg ha^-1^) | Chemical K_2_O  (kg ha^-1^) | Organic material  (kg ha^-1^) |
| --- | --- | --- | --- | --- | --- |
| FP | 975(B)+600(T)+60(U) | 234.6 | 205.5 | 302.3 | 0 |
| CFR | 750(SR) | 90.0 | 112.5 | 60.0 | 0 |
| OF | 750(SR) | 90.0 | 112.5 | 60.0 | 4500 (organic fertilizer) |
| BOF | 750(SR) | 90.0 | 112.5 | 60.0 | 4500 (bio-organic fertilizer) |
| Vm | 750(SR) | 90.0 | 112.5 | 60.0 | 5925 (vermicompost) |

Note: Farmer practice (FP); chemical fertilizer reduction (CFR); chemical fertilizer reduction combined with organic fertilizer (OF); chemical fertilizer reduction combined with bio-organic fertilizer (BOF); chemical fertilizer reduction combined with vermicompost (Vm). B, base compound fertilizer (N-P₂O₅-K₂O: 12-18-15, total nutrients ≥ 45%); T, topdressing compound fertilizer (N-P₂O₅-K₂O: 15-5-26, total nutrients ≥ 46%); U, urea (N content 46%); SR, slow-release compound fertilizer (N-P₂O₅-K₂O: 12-15-8, total nutrients ≥ 35%).


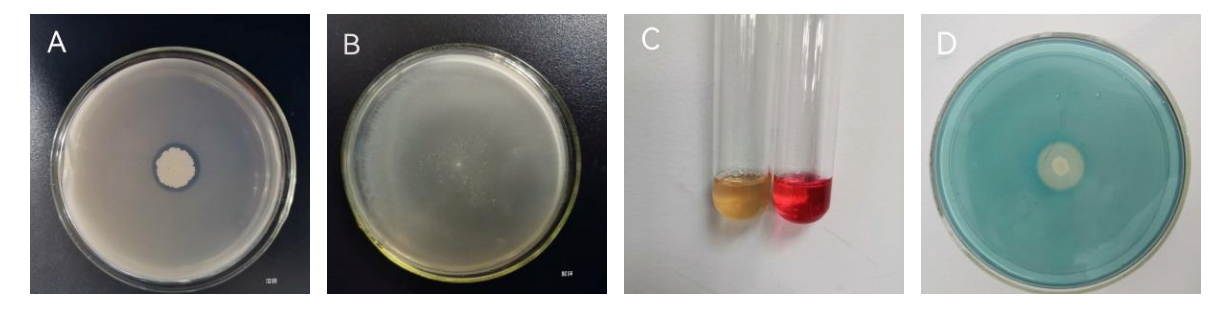


Figure S5. Phosphorus solubilization (A), potassium solubilization (B), IAA production (C), and siderophoregenesis (D) of strain C44

Note: (A) Phosphate solubilization: A transparent halo around the colony on inorganic phosphorus medium indicates phosphate-solubilizing capacity. (B) Potassium solubilization: No transparent halo was observed on silicate medium, suggesting a lack of potassium-solubilizing activity. (C) IAA production: The pink color reaction with Salkowski’s reagent confirms the synthesis of indole-3-acetic acid (IAA). (D) Siderophore secretion: The formation of an orange halo on CAS medium identifies strain C44 as a siderophore producer.

**Appendix C**

The appendix contains the chemical structures (Figure S6, mass spectrometry parameters (Figure S7), and chromatograms (Table S5) for the five analyte compounds in *A. membranaceus* samples, along with the UPLC-TQ-MS method validation parameters (Table S6) and recovery results (Table S7).


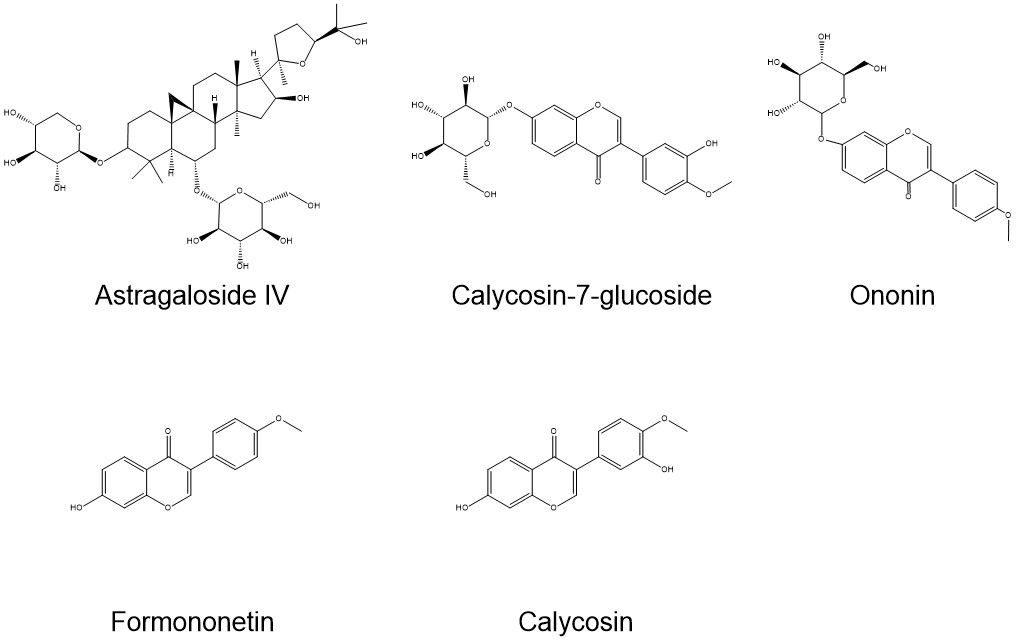


Figure S6. The structures of the five analyte compounds in *A. membranaceus* samples.


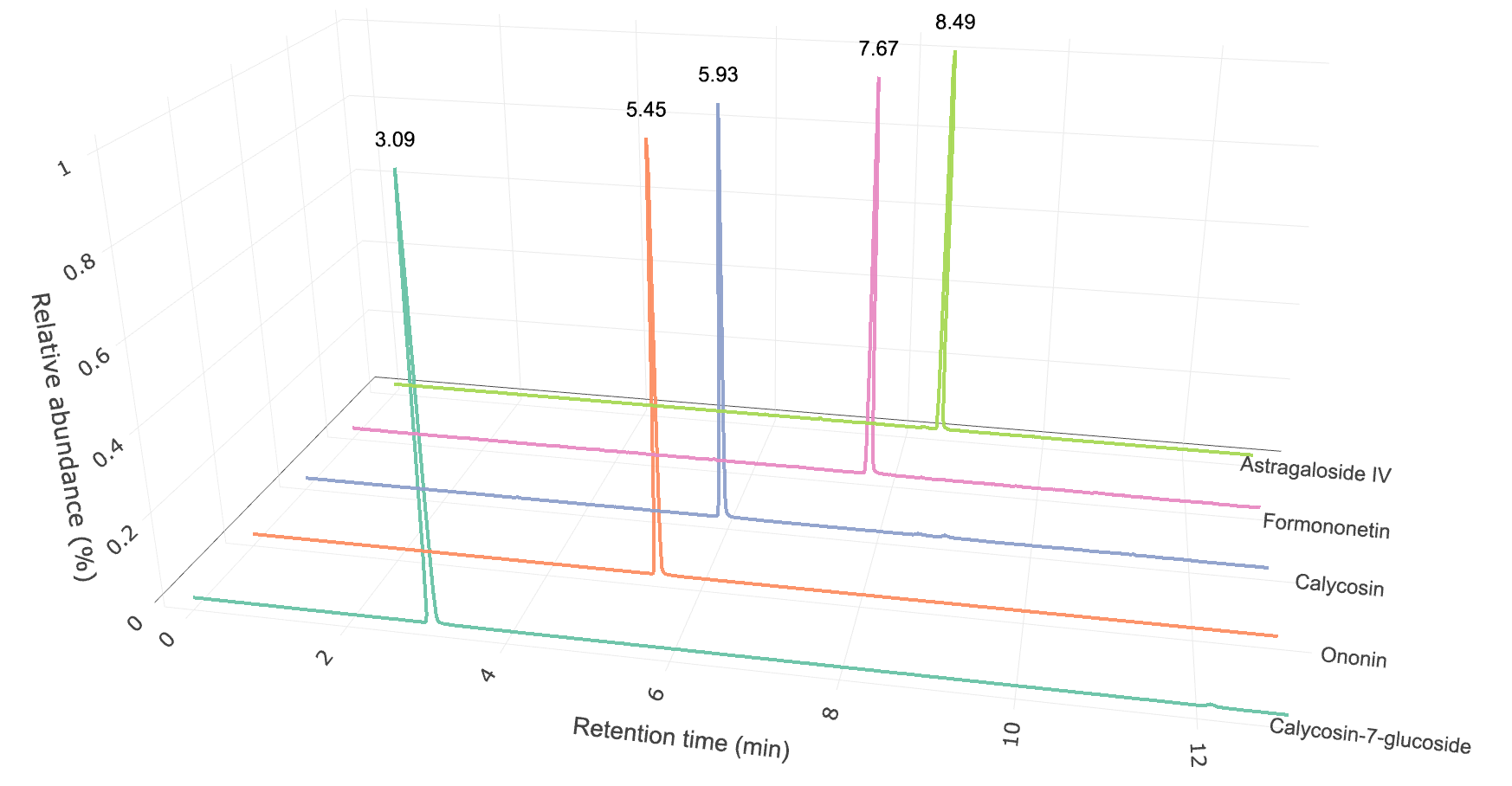


Figure S7. The mass spectrometry parameters of the five analyte compounds in *A. membranaceus* samples.

Table S5. The chromatograms of the five analyte compounds in *A. membranaceus* samples.

| Compound | Retention  Time  (min) | Ionization  Mode | Parent  Ion  (m z^-1^) | Daughter  Ion  (m z^-1^) | Cone  Voltage  (V) | Collision  Energy  (eV) |
| --- | --- | --- | --- | --- | --- | --- |
| Astragaloside IV | 8.49 | ESI- | 829.30 [M+HCOO]- | 45.00，829.30* | 30 | 15，35 |
| Calycosin-7-glucoside | 3.09 | ESI+ | 446.88 [M+H]+ | 269.80，284.90* | 2 | 16，40 |
| Ononin | 5.45 | ESI+ | 430.95 [M+H]+ | 196.90，268.90* | 18 | 14，68 |
| Formononetin | 7.67 | ESI+ | 268.63 [M+H]+ | 114.80，196.80* | 14 | 38，68 |
| Calycosin | 5.93 | ESI+ | 284.83 [M+H]+ | 213.00，269.90* | 8 | 20，36 |

Note: The ions marked with an asterisk (*) are the Quantitative Ions.

Table S6. Method validation parameters for the five analyte compounds in *A. membranaceus* samples using UPLC-TQ-MS.

| Compound | Regression  Equation | r | Range  (ng mL^-1^) | LOD  (ng mL^-1^) | LOQ  (ng mL^-1^) | Precision  (RSD, %) | Repeatability  (RSD, %) | Stability  (24 h, RSD, %) |
| --- | --- | --- | --- | --- | --- | --- | --- | --- |
| Astragaloside IV | y=4.931x+46.8 | 0.9980 | 10-1000 | 0.2505 | 0.8350 | 2.63 | 2.82 | 3.86 |
| Calycosin-7-glucoside | y=560.734x+9903.9 | 0.9995 | 10-1000 | 0.0207 | 0.0669 | 1.18 | 1.49 | 3.76 |
| Ononin | y=25.174x+209.6 | 0.9999 | 10-1000 | 0.3420 | 1.1401 | 1.61 | 2.81 | 2.87 |
| Formononetin | y=60.454x+1084.2 | 0.9996 | 10-1000 | 0.2700 | 0.8999 | 2.58 | 1.79 | 3.56 |
| Calycosin | y=246.711x+15045.1 | 0.9960 | 10-1000 | 0.2154 | 0.7178 | 2.88 | 1.42 | 4.63 |

Note: The results are expressed as the mean of replicates. Correlation coefficient (r); limit of detection (LOD); limit of quantification (LOQ); relative standard deviation (RSD).

Table S7. Recovery results for the five analyte compounds in *A. membranaceus* (n = 6).

| Compound | Sample Weight  (g) | Sample Content  (μg) | Spiked Amount  (μg) | Measured Amount  (μg) | Recovery  (%) | Average Recovery  (%) | RSD  (%) |
| --- | --- | --- | --- | --- | --- | --- | --- |
| Astragaloside IV | 1.000 2 | 2 389.577 8 | 2 400.0 | 4 926.559 7 | 102.86 | 100.35 | 4.75 |
|  | 1.000 2 | 2 389.577 8 | 2 400.0 | 5 189.986 5 | 108.36 |  |  |
|  | 1.000 1 | 2 389.338 9 | 2 400.0 | 4 831.964 0 | 100.89 |  |  |
|  | 1.000 2 | 2 389.577 8 | 2 400.0 | 4 650.680 0 | 97.10 |  |  |
|  | 1.000 2 | 2 389.577 8 | 2 400.0 | 4 659.301 3 | 97.28 |  |  |
|  | 1.000 1 | 2 389.338 9 | 2 400.0 | 4 577.650 1 | 95.58 |  |  |
| Calycosin-7-glucoside | 1.000 2 | 502.200 4 | 500.0 | 1 026.854 5 | 102.46 | 102.90 | 1.49 |
|  | 1.000 2 | 502.200 4 | 500.0 | 1 034.371 0 | 103.21 |  |  |
|  | 1.000 1 | 502.150 2 | 500.0 | 1 004.755 8 | 100.26 |  |  |
|  | 1.000 2 | 502.200 4 | 500.0 | 1 049.905 1 | 104.76 |  |  |
|  | 1.000 2 | 502.200 4 | 500.0 | 1 030.161 8 | 102.79 |  |  |
|  | 1.000 1 | 502.150 2 | 500.0 | 1 041.534 7 | 103.93 |  |  |
| Ononin | 1.000 2 | 113.122 6 | 100.0 | 213.186 5 | 100.03 | 99.21 | 2.80 |
|  | 1.000 2 | 113.122 6 | 100.0 | 212.461 9 | 99.69 |  |  |
|  | 1.000 1 | 113.111 3 | 100.0 | 217.565 3 | 102.09 |  |  |
|  | 1.000 2 | 113.122 6 | 100.0 | 202.359 9 | 94.95 |  |  |
|  | 1.000 2 | 113.122 6 | 100.0 | 216.617 8 | 101.64 |  |  |
|  | 1.000 1 | 113.111 3 | 100.0 | 206.355 7 | 96.83 |  |  |
| Formononetin | 1.000 2 | 12.402 5 | 10.0 | 21.168 1 | 94.49 | 97.24 | 3.64 |
|  | 1.000 2 | 12.402 5 | 10.0 | 21.369 7 | 95.39 |  |  |
|  | 1.000 1 | 12.401 2 | 10.0 | 21.325 9 | 95.20 |  |  |
|  | 1.000 2 | 12.402 5 | 10.0 | 22.962 6 | 102.50 |  |  |
|  | 1.000 2 | 12.402 5 | 10.0 | 21.251 0 | 94.86 |  |  |
|  | 1.000 1 | 12.401 2 | 10.0 | 22.625 2 | 101.00 |  |  |
| Calycosin | 1.000 2 | 179.235 8 | 180.0 | 334.376 7 | 93.08 | 99.38 | 3.36 |
|  | 1.000 2 | 179.235 8 | 180.0 | 367.426 4 | 102.28 |  |  |
|  | 1.000 1 | 179.217 9 | 180.0 | 360.511 1 | 100.36 |  |  |
|  | 1.000 2 | 179.235 8 | 180.0 | 362.109 7 | 100.80 |  |  |
|  | 1.000 2 | 179.235 8 | 180.0 | 353.595 8 | 98.43 |  |  |
|  | 1.000 1 | 179.217 9 | 180.0 | 364.067 3 | 101.35 |  |  |

Note: The values represent the accuracy of the method. The recovery was determined using the spike recovery test (n = 6). Relative standard deviation (RSD)

**Appendix D**

The appendix further includes the proportions of soil active carbon fractions to soil organic carbon (Figure S8) and the effect of chemical fertilizer reduction combined with organic amendments on soil carbon pool management index (Table S8).


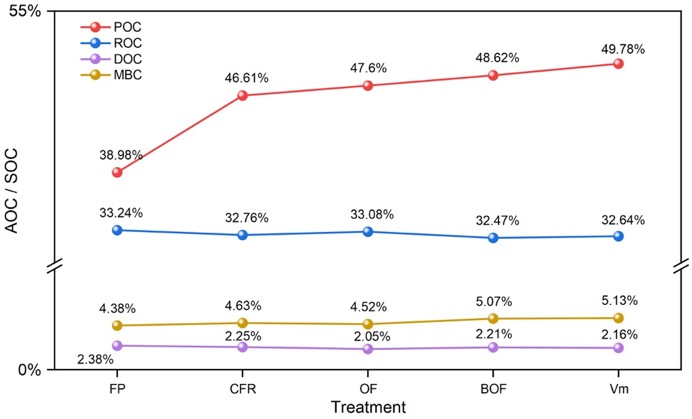


Figure S8. Proportions of soil active carbon fractions to soil organic carbon (SOC).

Note: Farmer practice (FP); chemical fertilizer reduction (CFR); chemical fertilizer reduction combined with organic fertilizer (OF); chemical fertilizer reduction combined with bio-organic fertilizer (BOF); chemical fertilizer reduction combined with vermicompost (Vm); active organic carbon (AOC); soil organic carbon (SOC); soil particulate organic carbon (POC); soil readily oxidizable organic carbon (ROC); soil dissolved organic carbon (DOC); soil microbial biomass carbon (MBC).

Table S8. Effect of chemical fertilizer reduction combined with organic amendments on soil carbon pool management index (CPMI).

| Treatment | SOC | CPI | ROC | NLC | L | LI | CPMI |
| --- | --- | --- | --- | --- | --- | --- | --- |
|  | (g kg^-1^) |  | (g kg^-1^) | (g kg^-1^) |  |  | (%) |
| CFR | 6.13c | 1.14 | 2.01 | 4.12 | 0.48 | 1.00 | 100 |
| FP | 5.54c | 1.04c | 1.84d | 3.70c | 0.48a | 1.02a | 92.37c |
| OF | 6.89a | 1.28a | 2.28b | 4.61ab | 0.49a | 1.03a | 113.98a |
| BOF | 7.21a | 1.34a | 2.34a | 4.87a | 0.49a | 1.05a | 115.93a |
| Vm | 6.67b | 1.24b | 2.18c | 4.49b | 0.50a | 1.05a | 108.07b |

Note: Different lowercase letters indicate significant differences between treatments at harvest (p < 0.05). Farmer practice (FP); chemical fertilizer reduction (CFR); chemical fertilizer reduction combined with organic fertilizer (OF); chemical fertilizer reduction combined with bio-organic fertilizer (BOF); chemical fertilizer reduction combined with vermicompost (Vm); soil organic carbon (SOC); carbon pool index (CPI); soil readily oxidizable organic carbon (ROC); non-labile carbon (NLC); carbon pool lability (L); lability index (LI); carbon pool management index (CPMI).
